# The Ketogenic Diet in the Prevention and Treatment of Hypertension (HTN)

**DOI:** 10.3390/biomedicines14081728

**Published:** 2026-07-31

**Authors:** Łukasz Rodzeń, Damian Dyńka, Mateusz Rodzeń, Hanna Karakuła-Juchnowicz, Dorota Łojko, Sebastian Kraszewski, Żaneta Grzywacz, Benjamin Bikman, Jen Unwin, David Unwin, Serafino Fazio

**Affiliations:** 1 Polish Society for Insulin Resistance Treatment (PTLI), 54-105 Wrocław, Poland; lukasz.rodzen@ptli.pl (Ł.R.); mateusz.rodzen@ptli.pl (M.R.);; 2 Rodzen Brothers Foundation, 64-234 Wieleń, Poland; 3 Institute of Health Sciences, Faculty of Medical and Health Sciences, University of Siedlce, 08-110 Siedlce, Poland; 4 I Department of Psychiatry, Psychotherapy and Early Intervention, Medical University of Lublin, 20-059 Lublin, Poland; 5 Department of Psychiatry, Poznan University of Medical Science, 60-572 Poznan, Poland; 6 Department of Biomedical Engineering, Faculty of Fundamental Problems of Technology, Wroclaw University of Science and Technology, 50-370 Wroclaw, Poland; 7 Department of Health Biosystems and Food Quality, Faculty of Physical Education and Physiotherapy, Opole University of Technology, 76 Prószkowska Street, 45-758 Opole, Poland; 8 Department of Cell Biology and Physiology, Brigham Young University, 3052 LSB, Provo, UT 84602, USA; 9 The Collaborative Health Community Foundation, Oxford OX2 9HZ, UK; 10 Faculty of Health Social Care and Medicine, Edge Hill University, Ormskirk L39 4QP, UK; 11 Department of Internal Medicine, School of Medicine, Federico II University, Via Sergio Pansini 5, 80131 Naples, Italy

**Keywords:** ketogenic diet (KD), hypertension, blood pressure, low carb, diet quality

## Abstract

Hypertension (HTN) is one of the greatest public health challenges of the 21st century. Its prevalence has reached alarming levels in recent decades. The search for effective prevention and treatment strategies includes lifestyle choices, such as dietary interventions. Although not applicable to everyone, particularly interesting in this context is the ketogenic diet (KD), known to clinicians and also applied in epilepsy treatment for over a century. Its possible beneficial effects are increasingly often reported in many other conditions, including HTN. The aim of the present study is to analyse the effect of a KD on blood pressure, and the mechanisms that may modulate that effect. Based on the available literature, key potential pathways of the influence of a KD on blood pressure regulation have been identified: (1) reduced body weight; (2) reduced visceral adipose tissue; (3) improved insulin sensitivity; (4) improved water and electrolyte balance; and (5) anti-inflammatory effect. Meta-analyses and randomised controlled trials consistently indicate that ketogenic dietary interventions are associated with reductions in blood pressure, although the magnitude of the effect varies considerably depending on the ketogenic diet model, study population, and comparator. In light of these observations, it has been found that in patients undergoing pharmacological treatment, it may be necessary to appropriately reduce antihypertensive medication dosage in advance. To maximise the hypotensive effect of a KD, the need to ensure an adequate supply of potassium, magnesium, and high-quality products, as well as proper hydration has been emphasised. Further studies are needed, with the effect of a KD on systolic and diastolic blood pressure as the primary endpoint, taking into account the role of the qualitative composition of the diet in the observed effects.

## 1. Introduction

Hypertension (HTN) is one of the key global health challenges of the 21st century and a leading cause of premature death worldwide [1,2]. An analysis of data from 200 countries and territories demonstrates the scale and gravity of the problem. It shows that in just 30 years (in the 30–79 age group), the prevalence of HTN nearly doubled—from 331 million women (6.24%) and 317 million men (5.98%) in 1990 to 626 million women (8.03%) and 652 million men (8.34%) in 2019, respectively [3]. These alarming statistics suggest that the preventive measures taken during the analysed period did not fully meet the expectations. This may indicate both the need to optimise the strategies used so far and to improve their acceptability and compliance by patients. One possible reason why the recommendations are rather ineffective may be the fact that they are not well-suited to clinical practice and to the way society is organised, which highlights the need for further improvements. Furthermore, it is necessary to explore new preventive and therapeutic strategies, and—given the importance of diet—various dietary strategies [4,5,6]. At the same time, the prevalence of insulin resistance is increasing worldwide, which, in addition to being the principal cause of type 2 diabetes, has been found to be closely associated with the development of cardiovascular diseases, and in particular, arterial hypertension [7,8].

The ketogenic diet (KD), known in clinical practice for over 100 years, is a highly viable option in this context. Its therapeutic properties were first described in 1921 in the context of treating drug-resistant epilepsy, a domain in which KDs have been successfully used ever since [9,10,11]. Over the last century, the scope of research into the potential clinical applications of the KD has expanded significantly. It now looks not only at other neurological disorders, such as Alzheimer’s disease, Parkinson’s disease, multiple sclerosis, migraine and Huntington’s disease [10,12], but also type 2 diabetes, for which KDs often outperform standard dietary strategies [13,14,15,16]. Other conditions of interest include metabolic (dysfunction)-associated fatty liver disease (MAFLD) [17], obesity [18,19], cancer [20], neuropsychiatric disorders [21,22,23], and cardiovascular diseases [24], including hypertension—which is the subject of this study. Furthermore, a KD improves insulin resistance with associated hyperinsulinaemia (chronically high blood insulin levels). In fact, severely limiting carbohydrates eliminates the major trigger for insulin release, helping to resolve hyperinsulinaemia (chronically high blood insulin levels).

The aim of this paper is to review and critically analyse the current scientific literature on the effect of a KD on blood pressure and to assess the grounds for using this particular diet as a dietary intervention in HTN patients. The paper presents the diet’s potential mechanisms of blood pressure regulation and attempts to organise and critically evaluate the existing data in this area. It also highlights the importance of a precise definition of the different variants of the ketogenic diet, which, according to the available evidence, may significantly affect the direction and intensity of its impact. The paper is based on a critical analysis of the current scientific literature on the impact of the KD on blood pressure parameters and the related physiological mechanisms. The selected sources consisted primarily of manuscripts published in English in high-impact journals, identified using the following keywords: ketogenic diet (KD), hypertension, blood pressure, low carb, diet quality, and insulin resistance.

## 2. Ketogenic Diet

The ketogenic diet (KD) can be defined as a nutritional strategy that aims to induce a state of ketosis, or increased production of ketone bodies such as β-hydroxybutyrate (the main ketone body), acetoacetate, and acetone [25]. At this basic level, this definition is generally acceptable. However, more detailed definitions and classifications of KD are open to numerous interpretations and ambiguities. Among other things, this is due to the fact that there are different types of ketosis: nutritional ketosis, which is the goal of dietary intervention, and non-nutritional ketosis, observed, for example, in starvation or ketoacidosis [10,26,27]. In the latter case, despite the increased ketone body concentrations, the desirable and stable metabolic state typical of a properly balanced KD is not achieved. Similar misunderstandings concern the classification of a KD simply as a high-fat and low-carbohydrate diet. While this is true in most cases, in some situations, this definition may be misleading. For example, a very low-calorie diet (VLCD, e.g., <800 kcal/day) can also accelerate ketogenesis, even though the supply of fat may be relatively low (e.g., 10 g) and carbohydrates may account for a significant share of the total energy supply [28,29,30,31,32,33]. In this case, ketogenesis is primarily a response to an energy deficit, rather than to a specific macronutrient composition. However, assuming that the KD is a normocaloric diet, and therefore does not cause the starvation effect or a major energy deficit, its qualitative composition is also essential, as discussed in Section Different KD Models—A Qualitative Comparison.

### Different KD Models—A Qualitative Comparison

Despite their common metabolic goal of inducing ketosis, the different KD models vary significantly in terms of quality and calorie content and will have slightly different effects (beneficial or not) on metabolic health. It is therefore reasonable to distinguish at least the following three KD models.

The first model focuses on high-quality foods and is normocaloric, which means that the energy supply is close to the body’s daily requirements. It is based on unprocessed foods and follows natural eating patterns as closely as possible. This diet is rich in fatty fish, eggs, good quality meat, seafood, offal, vegetables—with particular emphasis on green leafy vegetables (e.g., rocket, spinach, kale, lamb’s lettuce), as well as nuts (including walnuts, Brazil nuts, almonds), avocados, olive oil, natural cream butter, and a limited amount of low-sugar fruits (e.g., blueberries, raspberries, strawberries) [34,35,36,37,38,39]. This dietary model promotes the so-called nutritional ketosis, which is considered beneficial and desirable in the context of long-term metabolic health.

The second model is a low-quality, normocaloric ketogenic diet based predominantly on highly processed foods, including ready-made ketogenic snacks, sugar-free soft drinks, low-quality processed meats (e.g., pepperoni, fried bacon), highly refined vegetable oils (e.g., sunflower and corn oils), and ketogenic sweets. This dietary pattern is commonly referred to as “dirty keto” within the popular ketogenic community, although the term has no formal scientific definition [40,41,42]. While this dietary model may induce ketosis, its reliance on highly processed, nutrient-poor foods may limit its potential health benefits.

The third model is a group of very low-calorie diets, regardless of the quality of the ingredients. Given the significant energy deficit, this model borders on the starvation diet. Although adherence to this model may increase ketone body concentration, it is not conducive to achieving optimal nutritional ketosis. The fat content in this diet is often insufficient, and the carbohydrate content, although low in terms of the total energy supply, may be relatively high. This model usually leads to rapid weight loss and is not recommended as a long-term solution due to the risk of deficiencies and suboptimal effects on metabolism [19,43,44,45,46,47].

**Figure 1:**
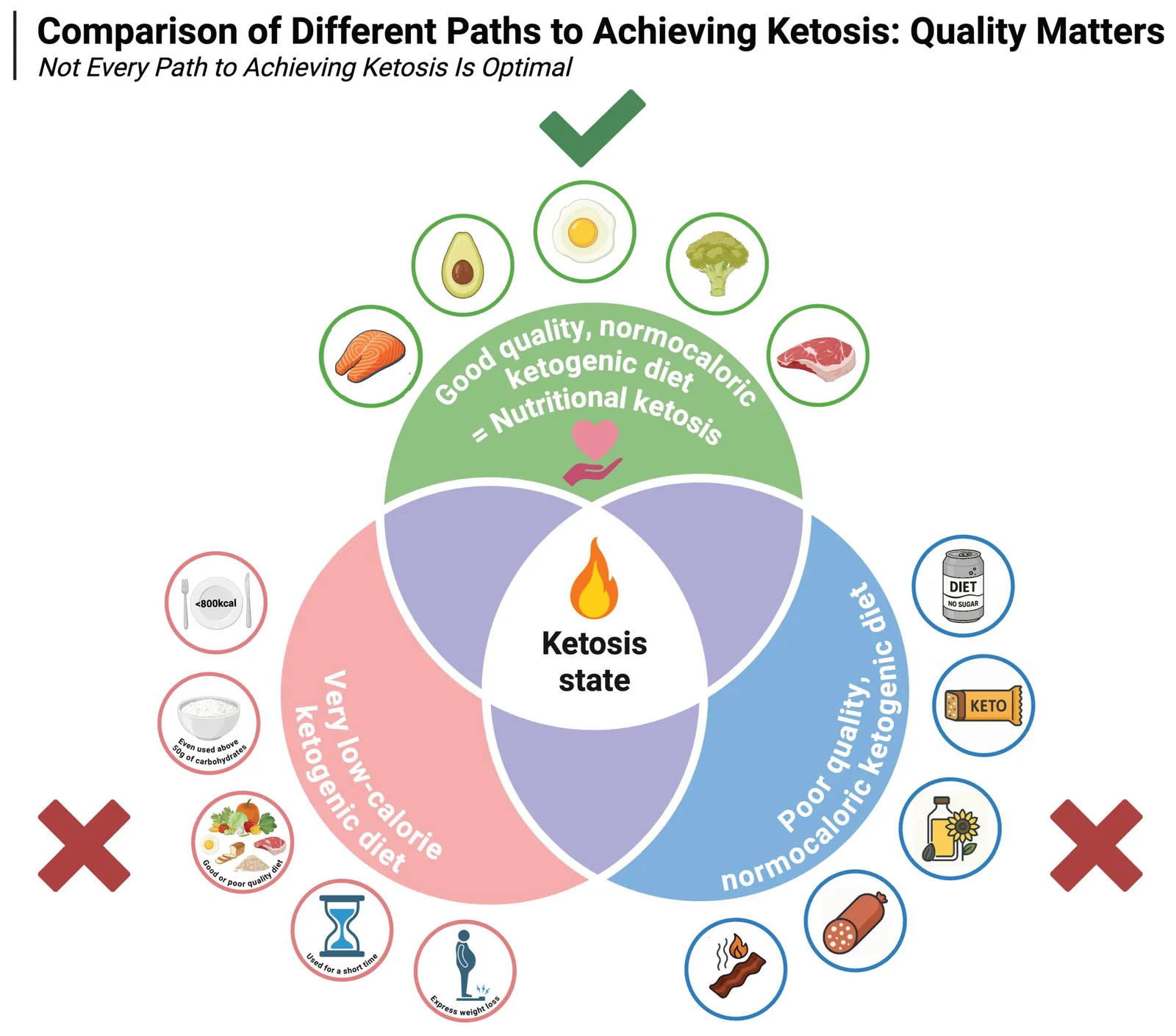
Distinguishing diet quality in achieving the desired state of nutritional ketosis. Created in BioRender. https://BioRender.com/7om6bxq (accessed on 30 June 2026).

With this in mind, the mechanism of inducing ketosis and the qualitative composition of the diet may be decisive. This should be clearly noted here, as these factors will directly impact blood pressure levels and the safety of the dietary treatment, as described in further sections of this paper. It should also be emphasised that the currently available clinical evidence is not evenly distributed across these dietary models. While studies evaluating very-low-calorie ketogenic diets (VLCKDs) and low-carbohydrate dietary interventions are relatively common, evidence specifically addressing high-quality normocaloric ketogenic diets in patients with hypertension remains limited. This distinction should be considered when interpreting the available clinical data presented in subsequent sections. A comparison of the quality of the different nutritional approaches to achieving ketosis is illustrated in Figure 1.

## 3. Hypertension (HTN)

Blood pressure, or the pressure applied to the walls of the arteries by blood flowing through them, is determined using two parameters: systolic blood pressure (SBP), achieved when blood is pumped from the heart; and diastolic blood pressure (DBP), or the pressure in the arteries during the diastolic phase of the heart when the ventricles fill with blood. Hypertension (HTN) is defined as blood pressure at or above 130/80 mmHg. The reference values for normal blood pressure are <120/80 mmHg [48].

Hypertension (HTN) is the leading cause of premature mortality worldwide. The most recent estimates show that the problem affects approximately 1.28 billion adults aged 30 to 79, of whom nearly 46% are unaware of their condition, while only one in five effectively controls their blood pressure [2]. HTN can lead to numerous organ complications, including cardiac (e.g., coronary artery atherosclerosis, ischaemic heart disease, left ventricular hypertrophy), cerebrovascular (e.g., ischaemic and haemorrhagic stroke), renal (up to end-stage renal failure), vascular (e.g., aortic aneurysm, peripheral artery disease), and ophthalmic (hypertensive retinopathy) [49].

HTN is divided into primary (also known as essential) and secondary, the former representing 90 to 95% of all HTN cases [50]. Primary HTN is usually idiopathic (although according to the latest findings, its aetiology is rather complex) and typically develops gradually with age. Secondary hypertension results from other diseases, a proven cause, the effects of medications, and usually resolves after their treatment or discontinuation [51,52].

The currently recommended treatment for hypertension includes both non-pharmacological and pharmacological approaches. Non-pharmacological methods include lifestyle and dietary modification, increased physical activity, giving up stimulants, and patient education. These recommendations should be followed by every patient with elevated blood pressure [51]. In the case of obesity (particularly visceral) and insulin resistance, which are responsible for as much as 65–75% of primary HTN, particular attention is paid to weight reduction [53]. Pharmacological treatment, in turn, includes a variety of medications, such as angiotensin-converting enzyme inhibitors (ACEis), angiotensin receptor blockers (ARBs), diuretics (usually thiazides), calcium channel blockers (CCBs) and beta-blockers (BBs) [51,54]. According to the WHO guidelines, the target blood pressure values for patients with hypertension without comorbidities are <140/90 mmHg. However, in patients at high cardiovascular risk, particularly those with diagnosed cardiovascular disease, diabetes, or chronic kidney disease, intensified treatment is recommended to keep systolic blood pressure levels below 130 mmHg [55,56].

## 4. Dietary Approaches to Stop Hypertension (DASH): A Standard Approach to Hypertension

DASH (short for Dietary Approaches to Stop Hypertension) is a diet developed in the 1990s and still recommended today [57], considered the standard dietary approach to treating hypertension. Its main principles include consuming 4–5 portions of vegetables, 4–5 portions of fruit, 6–8 portions of cereal products, 2–3 portions of low-fat dairy products and up to 6 small portions (approx. 30 g each) of meat, poultry or fish per day [58]. Carbohydrates should come mainly from whole grains, legumes and fruits with a low glycaemic index. The fats recommended in the diet are olive oil, avocados, nuts, flax seeds, and sea fish. Protein sources should primarily include legumes, soybeans, nuts and seeds. The recommended animal products include lean meat, fish, eggs, and low-fat dairy products [57].

**Figure 2:**
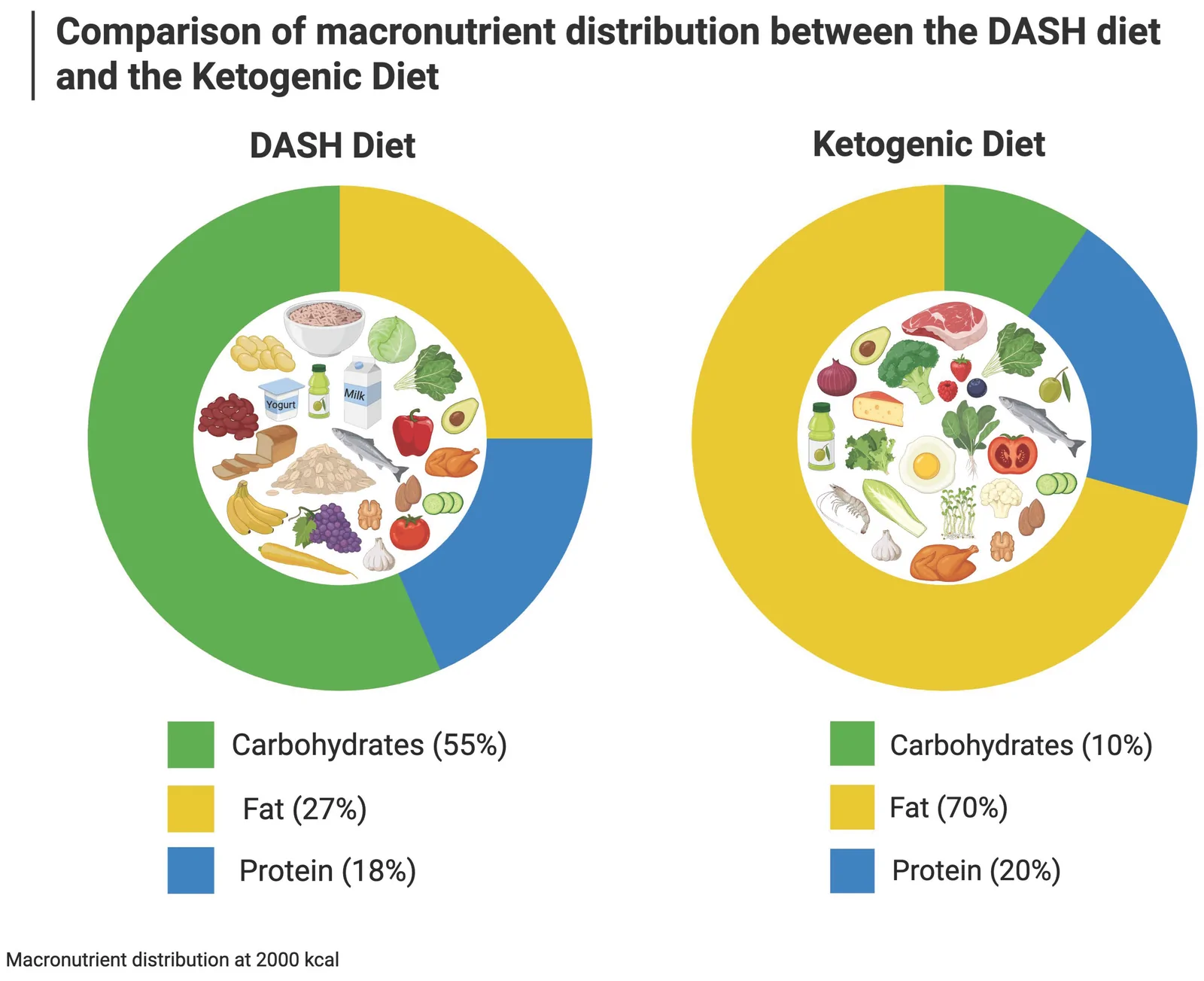
Comparison of macronutrient distribution in the DASH diet and the ketogenic diet. Created in BioRender. https://BioRender.com/88yabi4 (accessed on 30 June 2026).

The daily nutritional recommendations in the DASH diet include limiting sodium intake to 2300 mg. A further reduction to 1500 mg is believed to offer additional health benefits. In addition, the diet should supply approximately 4700 mg of potassium, 500 mg of magnesium, 1250 mg of calcium and approximately 30 g of dietary fibre. The recommended relative share of energy from individual macronutrients is as follows: 18% from protein, 27% from fat, and 55% from carbohydrates [59], which forms the basis of standard nutrition plans in this dietary model [60]. A comparison of the macronutrient distribution in the DASH diet and the ketogenic diet is shown in Figure 2.

It is estimated that dietary intervention based on the DASH model can effectively lower blood pressure [61,62]. As one meta-analysis demonstrated, adherence to this diet reduced systolic and diastolic blood pressure on average by 6.74 mmHg and 3.54 mmHg, respectively [63]. A 2025 study also showed that combining the DASH diet with sodium restriction may have additional benefits in reducing the risk of atherosclerotic cardiovascular disease (ASCVD) compared to the standard dietary pattern in the American population [64]. Importantly however, the relationship between the DASH diet and blood pressure values is not always statistically significant, also with regard to the occurrence of cardiovascular events [65,66].

Despite the numerous health benefits and widely documented effectiveness of the DASH diet, there is a growing body of research indicating that the ketogenic diet (KD) may have not only a comparable, but sometimes even more beneficial effect on blood pressure. One randomised clinical trial showed that compared to the DASH diet, a KD-based intervention resulted in a greater reduction in mean systolic blood pressure (−9.77 mmHg vs. −5.18 mmHg), a more significant improvement in glycated haemoglobin concentration (−0.35% vs. −0.14%), and a more pronounced body weight reduction [67]. There are a number of potential mechanisms (discussed in detail further on in this paper) through which the KD may affect blood pressure, showing a possible therapeutic advantage over the DASH model in selected aspects. Given the potentially greater effects of this nutritional approach compared to those of the currently recommended standards, it seems reasonable to further investigate this area, taking into account key and perhaps decisive nuances, in order to optimise HTN prevention and treatment. Interestingly, research on the DASH diet has focused primarily on its effect on blood pressure, while the ketogenic diet has been studied for over 100 years in the treatment of epilepsy and in many other physiological and clinical contexts.

However, it should be noted that the DASH diet was developed based on the contemporary views on the pathogenesis of hypertension and cardiovascular disease, and the randomised controlled trials available at the time generally covered relatively short periods of time [68,69]. Furthermore, given its elaborate and restrictive dietary requirements, the DASH model may be difficult to maintain in long-term clinical practice [70]. At the same time, the alternative models of hypertension pathophysiology developed in the recent decades better explain the observed effectiveness of metabolic ketosis in reducing blood pressure levels.

It should be emphasised, however, that the ketogenic diet is not a homogeneous dietary model. Its potential effects on blood pressure may depend not only on the degree of carbohydrate restriction, but also on the qualitative composition of dietary fat. For example, ketogenic diets enriched in monounsaturated fatty acids, particularly oleic acid from olive oil, avocados, and nuts, may influence endothelial function through mechanisms that differ from dietary patterns relying predominantly on saturated fatty acids, such as palmitic acid [71,72,73]. Consequently, Mediterranean-style ketogenic diets may differ from more conventional ketogenic dietary patterns in the mechanisms through which they influence cardiovascular health and blood pressure.

## 5. Materials and Methods

This paper is a narrative review of the literature, deliberately selected for the heterogeneity of the sources included, covering meta-analyses, randomised controlled trials, mechanistic studies, narrative reviews, case reports, observational studies, as well as clinical guidelines and expert recommendations. Searches were primarily conducted in the PubMed and Google Scholar databases. The initial search strategy included broad combinations of keywords related to the ketogenic diet (e.g., “ketogenic diet”, “ketosis”) and blood pressure, including “blood pressure” and “hypertension”. These were followed by more targeted search strategies, including terms related to potential pathophysiological mechanisms of hypertension, such as “insulin resistance and hypertension”, “insulin resistance and blood pressure”, and “body weight and blood pressure”, as well as their links to ketogenic diet intervention (e.g., “ketogenic diet and insulin resistance”, “ketogenic diet and body weight”). Similar search strategies were used for the other mechanisms. This approach enabled a comprehensive analysis of the literature not only in terms of the clinical impact of the ketogenic diet on hypertensive patients, but also in relation to the potential mechanisms behind this intervention.

## 6. Mechanisms and Target Areas of the Hypotensive Effect of the Ketogenic Diet (KD)

### 6.1. Body Weight Reduction

#### 6.1.1. Relationship Between Overweight/Obesity and Hypertension

Obesity and overweight are among the key public health challenges of the 21st century, with their worldwide prevalence steadily increasing. Since 1990, the prevalence of obesity has more than doubled in the adult population, while among young people, it has almost quadrupled. It is estimated that excess weight (including both overweight and obesity) affects approximately 2.5 billion adults, of whom as many as 890 million are obese. In the 5–19 age group, 390 million children and adolescents are overweight, including 160 million who are obese. In addition, 35 million children under the age of 5 meet the criteria for obesity [74,75]. This means that for the first time in history, there are more than one billion obese people in the world. Although the prevalence of overweight and obesity combined exceeds the prevalence of diagnosed hypertension, the number of people with obesity and hypertension are quite similar, as hypertension affects 1.28 billion people worldwide [76]). It turns out that both conditions are significantly related and often coexist. It has been shown that as many as 90–95% of cases of hypertension are primary [50], while obesity accounts for approximately 65–75% of cases of primary hypertension [53]. The two conditions are strongly and almost linearly correlated. The authors of one study observed a positive correlation between body weight and blood pressure. A 1 kg increase in body weight was associated with an increase in systolic blood pressure (SBP) of 0.725 mmHg and diastolic blood pressure (DBP) of 0.318 mmHg [77]. These figures emphasise the fundamental importance of weight control in the prevention and treatment of hypertension and highlight the importance of analysing the effectiveness of nutritional interventions aimed at reducing obesity—particularly visceral obesity, which is more strongly associated with hypertension risk than overall adiposity and cannot be adequately captured by BMI alone—as an integral part of antihypertensive therapy.

#### 6.1.2. Effect of the Ketogenic Diet on Body Weight Reduction

The effect of the ketogenic diet on body weight reduction is well-documented and widely recognised as one of its main therapeutic outcomes. It is therefore not surprising that most people follow this diet precisely for the purpose of losing weight [78,79,80]. There is a growing body of evidence indicating that there are certain areas in which the KD may have an advantage over dietary models with a greater supply of carbohydrates [19], and the DASH diet is clearly one of them [60]. The potential areas in which the KD may be superior to dietary models based on a higher carbohydrate intake (including the DASH diet) include its obvious effect on suppressing appetite and increasing the sense of satiety, which makes it easier to maintain an energy deficit in the long-term. In addition, rapid initial weight loss, resulting mainly from a reduction in glycogen and water stored in the body, can be an important motivating factor. Reduced daily fluctuations in blood glucose and insulin levels, which reduce the risk of hunger pangs and binge eating, are equally important. Furthermore, the KD may help manage chronic inflammation beyond the effect of weight loss alone, as well as reduce the need for pharmacological treatment of obesity by acting through similar mechanisms. Last but not least, the KD has a beneficial effect on mood and cognitive function, which may support adherence to the dietary recommendations. All of these aspects are described in detail in a 2025 paper [19].

The effectiveness of the ketogenic diet in body weight reduction has recently been confirmed in a number of meta-analyses and systematic reviews. For example, a 2025 systematic review with meta-analysis reported a significant improvement in body weight and body fat (in kg and %) in individuals consuming ≤50 g of carbohydrates per day. The authors explicitly concluded that overweight or obese adults should follow a ketogenic or low-carbohydrate diet for at least one month, with carbohydrate intake limited to ≤50 g/day [18]. Another systematic review, covering 110 randomised controlled trials, showed that every 10% reduction in carbohydrate intake was associated with a significant decrease in body weight, averaging 0.64 kg after 6 months and 1.15 kg after one year, with the effect being linear and sustained over time [81]. Similarly, a 2024 meta-analysis of overweight, obese, and polycystic ovary syndrome (PCOS) women showed that KD intervention led to a statistically significant reduction in several parameters, including body weight (mean −9.13 kg), waist circumference (average −7.62 cm), and fat mass (average −5.32 kg) compared to control groups [82]. Additionally, another study aggregating data from studies looking at overweight patients with type 2 diabetes confirmed the benefits of the ketogenic diet. The improvement in HbA1c, HDL, and triglycerides was accompanied by statistically significant weight loss (SMD = −5.63) and waist circumference reduction (SMD = −2.32) [83].

Importantly, the KD also shows an advantage in terms of weight loss in conditions where both the study and control groups are allowed to eat ad libitum. This may indicate that the KD is more likely to spontaneously generate an energy deficit, which seems to be related to its beneficial effect on satiety and appetite regulation. Similar effects have also been observed in studies where the total energy content of the diet was comparable between the study and the control group. This has been confirmed by a number of randomised controlled trials [84,85,86,87,88,89], aggregated and analysed in one of the latest papers [19].

Since weight loss has a well-documented effect on lowering blood pressure, and given the proven effectiveness of the ketogenic diet in reducing body weight, the available evidence is consistent with the hypothesis that weight loss is one of the key mechanisms through which the ketogenic diet may contribute to blood pressure reduction. Importantly, in this particular regard, the KD may outperform dietary models with a higher carbohydrate supply, as demonstrated above.

### 6.2. Reduced Insulin Resistance (IR)

#### 6.2.1. Relationship Between Insulin Resistance and Hypertension/Obesity

Obesity is often accompanied by insulin resistance (IR), another condition associated with increased overall mortality [90] and thus constituting a significant public health challenge. Despite the absence of clear diagnostic criteria and widespread screening, based on the HOMA-IR index, it has been estimated that IR may affect at least 40% of American adults aged 18–44 [91]. Global estimates suggest that the problem may affect up to 46.5% of the adult population [92]. However, since the sensitivity of the HOMA-IR is limited (the index is based solely on fasting glucose and insulin concentrations), the actual prevalence of insulin problems may be significantly higher. Another key aspect is that insulin resistance is a key pathophysiological mechanism in type 2 diabetes mellitus (T2DM), which accounts for approximately 90% of all cases of diabetes, affecting 10.5% of the global adult population [93]. Importantly, as many as 90% of people with type 2 diabetes are overweight [94], which further confirms the close relationship between obesity, insulin resistance and metabolic disorders. The same is true for hypertension. It has been known for years that at least 50% of patients with primary HTN show signs of insulin resistance, and given its low detection rate and increasing prevalence, the percentage of hypertension cases associated with IR may be significantly higher and include the majority of patients with primary hypertension [95]. For example, a 2023 study found that as many as 88.3% of patients with type 2 diabetes and normotension had developed insulin resistance compared to 92.5% of those with T2DM and hypertension [96]. A multivariate analysis showed that IR increases the risk of developing HTN by 150%, regardless of body weight [97]. This indicates that even slim people with a normal weight may be at a significantly greater risk of developing hypertension if suffering from insulin resistance.

Insulin resistance may contribute to the development of hypertension through a number of related mechanisms. In 2022, Kobayashi et al. [98] found that the accompanying hyperinsulinaemia (a condition leading to IR) promotes vascular endothelial dysfunction by reducing the bioavailability of nitric oxide, which disrupts the regulation of vascular tone. At the same time, excess insulin stimulates the proliferation of smooth muscle cells in vessel walls, making the walls thicker and changing their structure. In addition, if accompanied by insulin resistance, elevated insulin and aldosterone concentrations exacerbate processes that promote vascular stiffness. The cumulative effect of these disorders increases vascular resistance and accelerates the development of hypertension [99,100,101]. Furthermore, peaks in plasma insulin concentrations (even within the physiological range) activate the sympathetic nervous system. Although they do not in themselves increase blood pressure in healthy individuals whose blood vessels dilate in response to those changes [102,103], when combined with vascular stiffness in individuals with chronic insulin resistance, they cause a risk of elevated blood pressure. In addition, high insulin concentrations may increase blood pressure by increasing the activity of the renin–angiotensin–aldosterone system (RAAS), described in detail in Section 6.4.2.

#### 6.2.2. Effects of the Ketogenic Diet on Reducing Insulin Resistance

Reducing insulin resistance is one of the key beneficial effects of the ketogenic diet, resulting from a number of related mechanisms. Firstly, the KD is an effective weight loss strategy and promotes intuitive maintenance of an energy deficit [19], and weight loss is a recognised factor in improving tissue sensitivity to insulin. Numerous studies have demonstrated a relationship between excess body weight and elevated HOMA-IR, and between weight loss and insulin resistance remission [104]. A recent study showed that IR and hyperinsulinemia remission reached 100% among individuals who had lost more than 30% of their body weight [105]. Secondly, the glycaemic index of the ketogenic diet is very low, which in itself promotes improved insulin management. A low GI diet is an effective therapeutic strategy in type 2 diabetes, and on top of that, has an insulin-sensitising effect in non-diabetics. A 2025 meta-analysis confirmed that low GI diets significantly reduce HOMA-IR in non-diabetic adult populations, which may be important in the context of IR withdrawal and the prevention of T2DM development in people with insulin resistance [106]. Thirdly, in addition to its low GI, the KD’s effect on postprandial insulin concentration is smaller compared to carbohydrate-based diets. One randomised controlled trial compared glucose and insulin levels after a KD meal vs. a Mediterranean diet (MD) meal. Apart from a major difference in glucose levels (much higher after the MD meal), insulin levels after the MD meal increased from 40 ± 4 pmol/L to 497 ± 101 pmol/L after 20 min, reaching 190 ± 23 pmol/L after 180 min. After the KD meal, insulin concentration remained much more stable (increasing from 44 ± 5 pmol/L to 88 ± 12 pmol/L after 30 min and then decreasing to 48 ± 4 after 180 min) [107]. The results clearly indicate that low-carbohydrate meals, typical for the ketogenic diet, stimulate a significantly lower insulin response than mixed meals. It is not without reason that carbohydrates should be consumed at the end of a meal. In this way, glucose and insulin peaks after the meal are lower than when carbohydrates are consumed simultaneously with other macronutrients [108]. The ketogenic diet, with its marginal carbohydrate consumption, naturally minimises this postprandial surge, which is an important mechanism in reducing hyperinsulinemia. In addition, this low-carbohydrate model seems to have some anti-inflammatory properties [109], as discussed in detail in Section 6.5, which is important in the context of insulin resistance. IR, in turn, is associated with chronic inflammation, characterised by elevated concentrations of pro-inflammatory cytokines such as TNF-α and interleukins (e.g., IL-6), which disrupt insulin signalling by impairing the function of its receptors [110,111]. The KD has been shown to effectively reduce the levels of these inflammatory markers, including TNF-α, CRP, and IL-6 [109,112].

Since reduced insulin resistance has a beneficial effect on lowering blood pressure, and the KD offers a number of mechanisms that effectively alleviate IR, this is clearly one of the possible hypotensive mechanisms of action of this dietary model.

### 6.3. Reduced Visceral Adipose Tissue

#### 6.3.1. Relationship Between Visceral Adipose Tissue and Hypertension

Visceral adipose tissue (VAT) is a specific type of adipose tissue accumulated in the abdominal cavity, surrounding the internal organs. Its excessive amount, referred to as visceral obesity, is strongly associated with an increased risk of developing metabolic syndrome, chronic inflammation, dyslipidaemia, insulin resistance, certain cancers, as well as cardiovascular diseases, including hypertension, and with increased overall mortality [113,114,115]. A 2025 study showed a significantly higher incidence of hypertension in individuals in the top quartile of the Chinese visceral adiposity index (CVAI, an empirical index used to estimate visceral fat, developed and validated specifically for the Chinese population) compared to the bottom quartile (29.41% vs. 14.69%; *p* < 0.001). Multivariate logistic regression analysis revealed that participants in Q4 faced a 1.91-fold higher risk of developing hypertension than those in Q1, indicating a strong association between the severity of visceral obesity and the risk of hypertension [116]. Another study, which analysed the relationship between the visceral adiposity index (VAI) and the prevalence of hypertension in a representative population of American adults, demonstrated a significant association between these variables. It was found that the risk of hypertension increased by 17% with each 1-SD increase in VAI, and participants in the top VAI quartile (Q4) had a 95% higher risk of hypertension compared to those in the bottom quartile (Q1). These results indicate a strong, non-linear relationship between VAI and the prevalence of hypertension [117]. Correlations between blood pressure and indicators linked to excess visceral adipose tissue have also been confirmed by other publications [118,119]. It has been postulated that the relationship between obesity and hypertension (as described in Section 6.1) is at least partially mediated by fat distribution. An excessive amount of visceral fat has been associated with a higher risk of hypertension, greater than that caused by excess subcutaneous fat or fat located in the lower body [120].

#### 6.3.2. Effect of the Ketogenic Diet on Visceral Fat Reduction

Numerous studies have confirmed that the ketogenic diet is highly effective in reducing visceral fat. A randomised controlled trial involving semi-professional footballers showed that, despite being slightly higher in calories, the ketogenic diet led to a significant reduction in visceral fat (−63 g vs. −27 g), total fat mass (−1.55 kg vs. −0.92 kg), waist circumference (−4.19 cm vs. −1.38 cm), and extracellular water (−3.43% vs. 0.03%) compared to the so-called Western diet. Importantly, these results were achieved in just 4 weeks [121]. Similar findings were arrived at in another RCT, where an ad libitum ketogenic diet led to a marked reduction in visceral fat (from 688.9 ± 125.4 g to 592.4 ± 103.1 g) and total fat (from 12.0 ± 2.7 kg to 10.9 ± 2.2 kg) compared to a non-ketogenic diet (NKD) (changes: 658.0 ± 200.5 g to 624.2 ± 201.5 g and 11.3 ± 2.6 kg to 10.9 ± 2.7 kg, respectively). Importantly, only the participants in the ketogenic diet group reported weight loss (−1.4 kg), while the control group experienced a slight body weight increase (+0.9 kg) [122]. Another study on overweight adults showed an average visceral fat loss ranging from 13.93 ± 1.298 kg to 11.89 ± 1.061 kg after 3 months of following the KD. The authors also studied the effect of a 3-week course of the KD on healthy participants with normal body weight; also in this group, they noted a visceral tissue mass decrease, from 7.60 ± 0.65 kg to 6.78 ± 0.61 kg, i.e., by 0.82 kg (≈10.8%) [123]. In a study by Goss et al., obese elderly people followed a normocaloric ketogenic diet or low-fat diet for 8 weeks. The KD group showed a significantly greater mean total fat mass reduction (−9.7% vs. −2%), and the mean visceral fat loss was three times greater than in the low-fat diet group (−22.8% vs. −1.0%). These results suggest that the KD may be more effective in improving metabolic parameters in older adults, particularly through the reduction in visceral fat [124]. A randomised study involving 91 subjects compared the effects of a very low-carbohydrate, high-fat (VLCHF) diet, high-intensity interval training (HIIT), and a combination of the two (VLCHF + HIIT) on body composition parameters. After 12 weeks, a significant mean reduction in visceral adipose tissue (VAT) was observed only in the VLCHF (−142.0 g) and VLCHF + HIIT (−104.0 g) groups, along with reductions in body weight, total adipose tissue, trunk fat, and waist and hip circumferences. HIIT alone did not have a significant effect on these parameters, suggesting that the VLCHF diet, both as monotherapy and in combination with physical exercise, effectively improves body composition also by reducing VAT [125]. There are more studies demonstrating that the KD has a beneficial effect on visceral fat reduction [29,35,126], even when compared to a low-calorie, low-fat diet [127].

### 6.4. Effect of the Ketogenic Diet on Water and Electrolyte Balance

#### 6.4.1. Effect of Water and Electrolyte Balance on Blood Pressure Regulation

Water and electrolyte balance plays an important role in blood pressure regulation, primarily through its effect on circulating blood volume and vascular resistance—two key blood pressure determinants. Of particular importance in this context are sodium and potassium levels, as well as the complex interactions between the kidneys, endocrine systems and higher centres in the central nervous system [128,129,130].

Sodium, as the main cation of the extracellular space, plays a key role in maintaining its volume and osmolarity. An increase in plasma sodium concentration leads to an increase in osmolarity, which activates osmoreceptors in the hypothalamus and induces the secretion of vasopressin (also known as the antidiuretic hormone, ADH) from the posterior lobe of the pituitary gland [131,132]. ADH acts in two ways: on the one hand, acting through V2 receptors in the collecting ducts of the nephron, it increases water reabsorption, leading to an increase in plasma volume; on the other hand, at higher concentrations, it acts on V1a receptors in the smooth muscle of blood vessels, causing them to contract and thus increasing peripheral vascular resistance. Both these mechanisms increase blood pressure [133,134].

Potassium, on the other hand, is widely recognised as a hypotensive agent (a substance that lowers blood pressure) [135,136]. Among other things, it acts by affecting sodium transport in the nephrons. Increased potassium concentration in the tubular lumen limits sodium reabsorption through sodium channels, which increases its excretion in urine. As a result, plasma volume decreases, which in turn reduces blood pressure [137]. In addition, potassium has a vasodilatory effect—on the one hand, it activates potassium channels, leading to hyperpolarisation of the membrane, which limits the influx of calcium ions into the smooth muscles of the vessels, resulting in a decrease in vascular tone and a drop in blood pressure [138,139]. On the other hand, it can promote vasodilation by increasing the bioavailability of nitric oxide, which also reduces blood pressure [140]. In addition, high potassium concentrations inhibit renin secretion by the juxtaglomerular apparatus of the kidneys, which limits the production of angiotensin II, a powerful vasoconstrictor and stimulator of aldosterone secretion. Reduced aldosterone secretion limits sodium and water reabsorption in the renal tubules, which also contributes to lowering blood pressure [141,142,143].

#### 6.4.2. Effect of the Ketogenic Diet on Water and Electrolyte Balance

The ketogenic diet has a significant impact on water and electrolyte balance, especially in its early stages during the so-called keto-adaptation period [144]. One of the most prominent metabolic effects at this stage is increased diuresis, which results from two main mechanisms, both of which are triggered by a significant reduction in carbohydrate intake.

Firstly, limited carbohydrate intake rapidly depletes glycogen stores in skeletal muscles and the liver [144]. Glycogen, a macromolecular form of glucose storage, binds water at a ratio of approximately 3–4 g of water per 1 g of glycogen [145,146]. Therefore, a loss of 400–500 g of glycogen may release up to 2 litres of water, leading to its increased excretion by the kidneys. As is well-known, increased diuresis is accompanied by increased loss of electrolytes, including sodium and potassium [147].

The other key mechanism consists of decreasing insulin concentration in response to a significantly reduced carbohydrate supply [148]. Under physiological conditions, insulin stimulates sodium reabsorption in the renal tubules, mainly in the proximal tubule. When insulin availability decreases, this mechanism becomes inhibited, which triggers increased sodium excretion (natriuresis), which secondarily intensifies water loss (osmotic diuresis) and the ensuing loss of potassium and magnesium. All this combined leads to a decrease in plasma volume and a drop in blood pressure, which may also be associated with symptoms of the so-called “keto flu” [144,149,150].

Insulin also plays a role in regulating the renin–angiotensin–aldosterone (RAA) system, which is responsible for water and electrolyte homeostasis and maintaining blood pressure [151]. Hyperinsulinemia may cause RAA hyperactivity and thus sodium retention and ultimately hypertension. The authors of a 2024 study [152] even postulated that insulin resistance is a consequence of RAA hyperactivation, which is in turn provoked by excessive insulin secretion triggered by poor diet and/or other factors. Conversely, normalising insulin secretion, primarily through dietary modifications, appears to be crucial in the prevention and treatment of insulin resistance, diabetic complications, and hypertension itself.

With this in mind, the effect of the KD on insulin, glycogen, electrolyte, and fluid levels in the body may be an important factor contributing to blood pressure reduction. It is important to ensure a higher intake of potassium and magnesium, especially in the initial phase (when they are lost due to increased diuresis), in order to sustain the beneficial hypotensive effects of the KD triggered by changes in the concentrations of these electrolytes.

### 6.5. Reducing Inflammation

#### 6.5.1. Relationship Between Inflammation and Hypertension

Inflammation is closely related to the development and progression of hypertension. It is known that higher levels of CRP (C-reactive protein), hs-CRP (high-sensitivity C-reactive protein) and IL-6 (interleukin 6)—all of which are indicators of inflammation—are associated with an increased risk of developing hypertension, which grows linearly with the increase in inflammatory markers (even in low- and medium-risk groups), as demonstrated in one meta-analysis [153]. Conversely, blocking inflammatory pathways may be helpful in preventing hypertension [154]. A growing body of evidence indicates that both innate and acquired immunity play an important role in the pathogenesis of hypertension. In HTN, activation of inflammatory pathways promotes oxidative stress and endothelial dysfunction, leading to activation of the RAA and sympathetic systems and impaired renal sodium excretion. This inflammatory-oxidative cascade weakens the bioavailability of nitric oxide, intensifies the activation of the RAA and sympathetic systems, and reduces the kidneys’ ability to excrete sodium at normal blood pressure levels. In practice, this means that maintaining a neutral sodium balance requires higher blood pressure values, and the kidneys less effectively increase sodium excretion in response to increased blood pressure, which promotes sodium retention, vascular damage, and the perpetuation of hypertension [155]. Zhang et al. [156] pointed out that innate immune cells such as M1 macrophages, neutrophils, and dendritic cells secrete pro-inflammatory cytokines, while T lymphocytes, through the production of IFN-γ and IL-17, exacerbate oxidative stress and endothelial dysfunction. Conversely, regulatory T lymphocytes and M2 macrophages may have a protective effect. However, the authors point out that despite the availability of data, the inflammatory mechanisms underlying hypertension remain complex and not fully understood. Studies have confirmed that the NLRP3 inflammasome may play a leading role in hypertensive inflammation. Its mechanism of action involves both the central nervous system and peripheral modulation of inflammatory pathways. Activation of NLRP3 leads to the release of pro-inflammatory cytokines such as IL-1β and IL-18, which exacerbates oxidative stress, impairs endothelial function and promotes vasoconstriction, ultimately raising blood pressure [157,158]. This may be confirmed by the fact that pharmacological suppression of NLRP3 is an effective strategy for lowering blood pressure [159]. Hence, the inflammatory process may play an important role in the pathogenesis of hypertension, including through oxidative stress and endothelial dysfunction in the inflammatory cascade. One study also emphasised the importance of factors such as ageing and the action of aldosterone, which may promote the persistence of inflammation and the progression of hypertension [160]. The authors considered the link between inflammation and HTN to be so significant that they suggested the potential role of anti-inflammatory drugs in treating hypertension.

#### 6.5.2. Effect of the Ketogenic Diet on Inflammation

Anti-inflammatory action of the ketogenic diet is one of its key characteristics mentioned in a number of scientific papers [109,112,161,162].

Firstly, as mentioned in the previous section, it is known that the activation of the NLRP3 inflammasome increases the production of pro-inflammatory cytokines and exacerbates inflammation. It is also associated with the development of hypertension [157,158]. Meanwhile, β-hydroxybutyrate (BHB), the main ketone body produced in individuals following the KD, may inhibit its action. For example, studies have shown that unlike other ketones and short-chain fatty acids, BHB reduces caspase-1 activation and the secretion of pro-inflammatory cytokines IL-1β and IL-18, both in human monocytes and animal models. This mechanism is independent of classic pathways regulated by starvation, such as AMPK, ROS, or autophagy. The authors of one paper suggest that the beneficial effects of the ketogenic diet may be partly owed to the direct suppression of NLRP3 inflammasome activity by BHB [163]. Importantly, certain parameters of inflammation can be reduced immediately (for instance, by the diet’s effect on the NLRP3 inflammasome). In one study, these parameters were reduced in just 3 days. Its authors explicitly suggest that an isocaloric ketogenic diet followed for 3 days is a promising approach to improving metabolic and inflammatory status [164]. Considering that pharmacological inhibition of the NLRP3 inflammasome (as confirmed by preliminary results of studies on this type of medication) reduces inflammation and lowers blood pressure [159], the ketogenic diet may be its natural equivalent, exhibiting a similar mechanism of action through the endogenous inhibition of inflammasome activity, without the possible side effects associated with pharmacotherapy.

Secondly, the ketogenic diet restricts the intake of simple sugars, known to be pro-inflammatory. Their higher intake is correlated with hypertension and other cardiovascular diseases (CVD) [165,166,167]. Restricted intake of simple sugars is in itself one of the important mechanisms behind the anti-inflammatory effect of the KD, considered beneficial in the context of CVD [24]. A low glycaemic index (GI) diet may lower blood pressure compared to higher GI dietary models [168]. At the same time, it is known that the KD is characterised by a minimal GI, even compared to a well-composed Mediterranean diet, which nevertheless triggers a greater release of glucose and insulin when compared to the KD [107]. The ketogenic diet has an even lower glycaemic index than diets commonly described as low-glycaemic, which may explain its more beneficial effect on carbohydrate metabolism parameters, including glucose and insulin levels. A study by Westman et al. demonstrated that a diet with a lower carbohydrate content led to greater improvement in glycaemic control and more frequent reduction or complete discontinuation of antidiabetic drugs compared to a low glycaemic index diet [169]. Importantly, the glycaemic index of a diet depends primarily on the quantity and quality of carbohydrates consumed [170,171]. In the KD, their supply is not only significantly reduced, but also confined to low GI foods, mainly non-starchy vegetables and nuts. At the same time, simple sugars, which have the greatest impact on postprandial glycaemia levels, are almost absent from this diet [36]. In comparison, standard dietary recommendations allow up to 10% of energy to come from simple sugars, which in a 2000-kcal diet corresponds to 50 g of sugar per day, or as much as 12 level teaspoons of white sugar [172,173]. As a result, official dietary recommendations allow for the consumption of simple sugars in an amount that corresponds to the maximum total carbohydrate intake in a ketogenic diet, the source of these carbohydrates in the KD being mainly non-starchy vegetables and nuts [36].

A 2025 meta-analysis demonstrated that each 1-unit increase in the Dietary Inflammatory Index (DII) score was associated with a 4% increase in the incidence of HTN. This finding is another piece of evidence that a pro-inflammatory diet increases the risk of hypertension. Therefore, limiting pro-inflammatory foods in the diet may be beneficial in the prevention and control of HTN [174]. With this in mind, the anti-inflammatory nature of a well-composed KD may be an important factor in lowering blood pressure.

**Figure 3:**
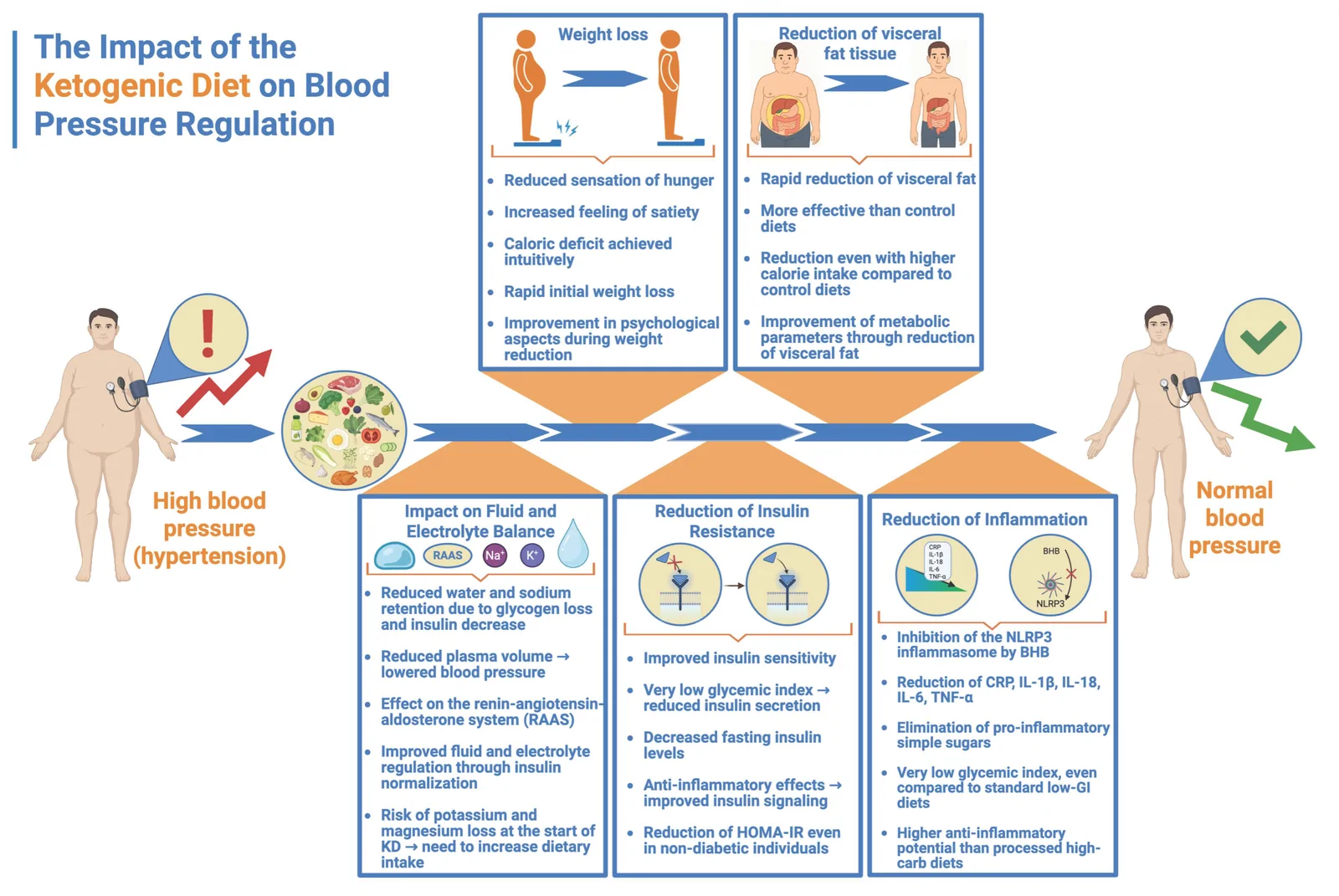
Mechanisms and target areas of the hypotensive effect of the ketogenic diet (KD). Created in BioRender. https://BioRender.com/rnwyhqn (accessed on 30 June 2026). Abbreviations: RAAS, renin–angiotensin–aldosterone system; Na^+^, sodium; K^+^, potassium; BHB, β-hydroxybutyrate; CRP, C-reactive protein; IL-1β, interleukin-1 beta; IL-6, interleukin-6; IL-8, interleukin-8; TNF-α, tumor necrosis factor alpha; NLRP3, NOD-like receptor family pyrin domain-containing 3; HOMA-IR, homeostatic model assessment of insulin resistance; KD, ketogenic diet.

It should be emphasised that the mechanisms described above are closely interconnected rather than acting independently. For example, weight loss induced by the ketogenic diet contributes to reductions in visceral adipose tissue, which in turn improves insulin sensitivity and attenuates chronic low-grade inflammation. Improved insulin sensitivity may also reduce hyperinsulinaemia-mediated activation of the renin–angiotensin–aldosterone system, thereby influencing sodium handling, fluid balance, and blood pressure regulation. At the same time, the anti-inflammatory effects of the ketogenic diet may further improve endothelial function and vascular homeostasis. Therefore, the antihypertensive effects of the ketogenic diet are most likely the result of multiple complementary and interacting physiological mechanisms rather than any single isolated pathway. Figure 3 is a graphical representation of the hypotensive effect of the KD described above.

## 7. Ketogenic Diet and Blood Pressure—Analysis of Evidence from Clinical Trials

### 7.1. Ketogenic Diet and Blood Pressure in Humans—Results of Meta-Analyses and Systematic Reviews

There are already a number of meta-analyses and systematic reviews—considered top-quality scientific evidence—looking at the effect of the KD on blood pressure in humans. The first meta-analysis [175], covering 29 clinical trials involving 2359 patients with type 2 diabetes, assessed the impact of very low-calorie ketogenic diets (VLCKD) on cardiovascular risk factors. VLCKDs were significantly more effective in reducing systolic (WMD = −2.85 mmHg) and diastolic (WMD = −1.40 mmHg) blood pressure compared to control diets, with a stronger effect on SBP in the subgroup of individuals with severe obesity (BMI > 35 kg/m^2^) (WMD = −3.15 mmHg). This finding is consistent with the well-documented relationship between visceral adiposity and hypertension risk (discussed in Section 6.3), suggesting that the greater hypotensive effect observed in this subgroup may be at least partly mediated by higher visceral fat burden rather than overall body weight per se. It should be acknowledged, however, that the currently available clinical evidence does not always allow the effects of nutritional ketosis to be clearly distinguished from those attributable to caloric restriction, weight loss, and the accompanying metabolic improvements. Therefore, the antihypertensive effects observed in ketogenic dietary interventions are likely to result from the combined contribution of these factors rather than ketosis alone. In addition, VLCKDs offered greater reductions in fasting glucose (−11.68 mg/dL), HbA1c (−0.29), insulin (−1.45), HOMA-IR (−0.71), and triglycerides (−17.95). All results were statistically significant (*p* < 0.05). Another meta-analysis [176], comprising 27 randomised studies involving 1278 participants, also assessed the effect of the ketogenic diet (vs. control diets) on cardiovascular risk factors. A significant reduction in diastolic blood pressure (WMD = −1.41 mmHg) was demonstrated, with no significant differences in systolic blood pressure. This was accompanied by greater reductions in triglyceride concentration (−0.20 mmol/L), glucose (−0.18 mmol/L), insulin (−8.32 pmol/L), and body weight (−2.59 kg), among other parameters, with a simultaneous increase in total cholesterol (TC) (+0.36 mmol/L), HDL (+0.16 mmol/L), and LDL (+0.35 mmol/L). Despite the increase in TC and LDL, the authors concluded that the ketogenic diet may be beneficial in managing blood pressure, body weight, and glycaemia. However, these findings should be interpreted with caution, particularly in patients with hypertension who already have an elevated cardiovascular risk profile. In clinical practice, the potential benefits of blood pressure reduction should therefore be balanced against changes in the lipid profile and assessed on an individual basis. Importantly however, not all meta-analyses confirm the KD’s significant effect on blood pressure, and the results largely depend on the methodology used. In one such analysis [177], covering 23 randomised clinical trials involving 1664 participants, only a small, statistically insignificant reduction in systolic (WMD: −0.87 mmHg) and diastolic (WMD: −0.11 mmHg) blood pressure was observed. Subgroup analyses did not provide additional information, and a non-linear dose–response analysis showed no association between the percentage of fat in the ketogenic diet and blood pressure levels. Slightly different results were arrived at in the meta-analysis by Jayedi et al. [178], which covered 50 clinical trials involving 4291 patients with type 2 diabetes. This analysis showed a significant relationship between dietary carbohydrate content and systolic blood pressure (SBP) and other cardiometabolic parameters. Compared to a diet with 65% of energy coming from carbohydrates, a 10% reduction in carbohydrate intake was associated with a mean gradual decrease in SBP: for 55% −2.37 mmHg (−6.99 to −2.34), 45% −4.59 mmHg (−8.21 to −0.97), 40% −5.44 mmHg (−9.41 to −1.47), 35% −6.17 mmHg (−9.87 to −2.12), 30% −6.86 mmHg (−10.80 to −3.12), 25% −7.56 mmHg (−10.98 to −3.29), 20% −8.25 mmHg (−12.0 to −4.25), 15% −8.95 mmHg (−12.25 to −4.50), and 10% −9.64 mmHg (−12.6 to −6.7). Carbohydrate restriction was also associated with a linear reduction in HbA1c (by an average of −0.20%), fasting blood glucose (−0.34 mmol/L), and body weight (−1.44 kg). These results suggest that the greater the reduction in carbohydrate intake, the lower both the systolic blood pressure and most of the cardiometabolic parameters analysed. The most pronounced benefits were observed with 10% of energy coming from carbohydrates, which is exactly what the ketogenic diet offers. Another meta-analysis from 2022 [179] assessed the level of scientific evidence on the benefits and harms of carbohydrate-restricted diets and intermittent fasting (IF) in order to propose reasonable recommendations. Very low-carbohydrate diets (VLCDs) reduced the mean systolic blood pressure by −4.97 mmHg vs. −3.0 mmHg in the control groups (mean difference: −1.97 (−3.68 to −0.25) over 6 months, and this difference was even greater = −8.1 mmHg (95% CI, −13.35 to −2.85) over periods exceeding 6 months (up to 1 year). However, no significant effect on diastolic blood pressure (−2.78 mmHg vs. −2.1 mmHg in control groups) was observed in overweight or obese individuals, as was the case in adults with T2DM (−1.36 mmHg SBP and −1.12 mmHg DBP in VLCD vs. 1.7 mmHg and −2.5 mmHg in control groups). At the same time, VLCD groups reported an almost twofold greater reduction in body weight (−7.42 kg vs. −3.75 kg in the control group), fat mass (−7.81 kg vs. −4.8 kg), and waist circumference (−8.81 cm vs. −4.7 cm), as well as a reduction in fat-free mass (−1.35 kg vs. −0.3 kg), HbA1c (−0.42 vs. −0.15), and fasting insulin (−2.92 μU/mL vs. −1.55 μU/mL), both in overweight/obese individuals, as well as in individuals with type 2 diabetes (HbA1C −0.56 vs. −0.2; HOMA-IR −1.52 vs. −0.45; fasting glucose −26.84 mg/dL vs. −17.2 mg/dL; body weight −7.24 kg vs. −3.4 kg). In both groups of patients, VLCD resulted in a greater reduction in TG and an increase in HDL and LDL. Another meta-analysis [180] aimed at evaluating the efficacy and safety of a very low-calorie ketogenic diet (VLCKD) in overweight and obese individuals. The analysis covered 12 studies with a total of 801 patients. Although only four of these studies (involving 199 individuals) reported blood pressure data, a statistically significant reduction in systolic blood pressure (−8.5 mmHg, ranging from −11.4 to −5.6) and diastolic blood pressure (−7.2 mmHg, ranging from−8.9 to −5.5) in the VLCKD group was demonstrated. In addition, this intervention was associated with significant body weight loss—an average of 10.0 kg for protocols with a ketogenic phase of ≤4 weeks and 15.6 kg for longer interventions. There was also a significant reduction in waist circumference (−12.6 cm), HbA1c (−0.7%), total cholesterol (−28 mg/dL), triglycerides (−30 mg/dL), and liver enzyme activity: AST (−7 U/L), ALT (−8 U/L), and GGT (−8 U/L), among other parameters. Another meta-analysis [181] assessed whether a very low-carbohydrate ketogenic diet (VLCKD; ≤50 g carbohydrates/day) offered more beneficial long-term effects in weight reduction and improvement in cardiovascular risk factors compared to a conventional low-fat diet (LFD; <30% energy from fat, with a calorie deficit). The VLCKD group reported a significantly greater reduction in diastolic blood pressure (WMD = −1.43 mmHg; 95% CI: −2.49 to −0.37), while the reduction in systolic blood pressure (WMD = −1.47 mmHg; 95% CI: −3.44 to 0.50) did not differ significantly from that in the LFD group. The VLCKD group also showed greater body weight reduction (WMD = −0.91 kg), lower triglyceride levels (WMD = −0.18 mmol/L), and higher HDL-C (WMD = +0.09 mmol/L) and LDL-C (WMD = +0.12 mmol/L) levels. A summary of the examined meta-analyses and systematic reviews is presented in Table 1.

### 7.2. Ketogenic Diets and Blood Pressure in Humans—Results of Randomised Controlled Trials (RCT)

The effect of the ketogenic diet on blood pressure has been assessed in a large number of randomised controlled trials (RCTs), considered the gold standard in intervention studies. A 2024 study [182] compared the newly developed Healthy Ketogenic Diet (HKD) against an energy-restricted diet (ERD) in terms of weight loss and metabolic parameters in obese adults. After 6 months of following the HKD, a significantly greater reduction in systolic blood pressure was observed compared to the ERD group (−7.7 ± 8.9 mmHg vs. −2.6 ± 12.2 mmHg; *p* = 0.005). The diastolic blood pressure reduction, while not statistically significant, was also greater (−3.7 ± 6.5 mmHg vs. −2.4 ± 7.7 mmHg). The HKD group also reported a significantly greater body weight loss (−7.8 ± 5.2 kg vs. −4.2 ± 5.6 kg; *p* = 0.01) and more favourable metabolic changes, including reduced HbA1c (−0.3 ± 0.3% vs. −0.1 ± 0.2%; *p* = 0.008) and AST activity (−7.6 ± 15.5 IU/L vs. +0.6 ± 11.5 IU/L; *p* = 0.01). Importantly, no increase in LDL cholesterol levels was observed in either group. The authors concluded that the HKD was more effective than the ERD in reducing body weight and improving cardiometabolic parameters without increasing LDL levels, which makes the HKD a viable candidate as a therapeutic intervention in obese patients. In another randomised study from 2024 [183], two versions of the Asian ketogenic diet (one based on whole eggs (Yolk-AKD) and one without egg yolks (White-AKD)) were compared against a balanced low-calorie diet (BLC) in patients with metabolic syndrome. In a long-term follow-up (from week 35 to week 52), ketogenic diets were shown to be more effective in reducing blood pressure compared to the control diet. At week 35, the mean systolic blood pressure (SBP) in the BLC group was 132.2 ± 2.4 mmHg and the diastolic blood pressure (DBP) was 88.7 ± 1.5 mmHg, while at week 52, they grew to 134.2 ± 2.1 mmHg and 91.7 ± 1.3 mmHg, respectively. For comparison, in the White-AKD group, SBP values were 121.9 ± 2.2 mmHg (week 35) and 123.2 ± 1.9 mmHg (week 52), and DBP values were 81.5 ± 1.4 mmHg and 82.3 ± 1.2 mmHg. In the Yolk-AKD group, SBP was 126.3 ± 2.1 mmHg (week 35) and 127.7 ± 1.8 mmHg (week 52), and DBP was 87.2 ± 1.4 mmHg and 87.6 ± 1.1 mmHg, respectively. In addition, both ketogenic diet groups showed more favourable changes in glucose tolerance, lipid profile, and liver function compared to the control group. Another RCT [184], including 29 overweight and obese individuals and covering a shorter period (10 days), compared three isocaloric dietary interventions: a ketogenic diet, a ketogenic diet with added ketone esters, and a standard control diet. In this case, ketogenic diet groups did not perform better. In the standard ketogenic diet group, SBP changed from 125 ± 3 to 125 ± 2 mmHg (*p* = 0.867) and DBP changed from 78 ± 3 to 77 ± 3 mmHg (*p* = 0.773); in the group following the ketogenic diet with ketone ester supplementation, SBP changed from 124 ± 2 to 121 ± 3 mmHg (*p* = 0.463) and DBP from 76 ± 2 to 76 ± 2 mmHg *p* = 0.976. In the standard diet group, SBP changed from 134 ± 2 mmHg to 123 ± 4 mmHg (*p* = 0.048) and DBP from 80 ± 3 to 73 ± 3 mmHg (*p* = 0.158). In addition to the KD’s insignificant effect on blood pressure, it was shown that the sensitivity of the whole body (muscles), liver and adipose tissue to insulin remained unchanged in all 3 groups, as did the plasma lipid profile. In another RCT comparing the effect of the ketogenic diet (KD) with varying sodium content versus that of the low-fat diet (LFD) on the RAA system in overweight and obese adults, no significant differences in blood pressure were found between the groups [185]. In the Ketogenic Diet: Ketogenic Supplement group, the differences in systolic blood pressure averaged −0.54 mmHg after 2 weeks, 2.62 mmHg after 4 weeks, and −6.87 mmHg after 6 weeks. In the Ketogenic Diet: Low-Fat Diet group, they were −6.34 mmHg, −4.51 mmHg and −6.21 mmHg, respectively, whereas in the Ketogenic Supplement: Low-Fat Diet group, they were −5.80 mmHg, −7.13 mmHg and 0.66 mmHg. With regard to diastolic blood pressure, in the Ketogenic Diet: Ketogenic Supplement group, the changes were: 3.31 mmHg after 2 weeks, 2.31 mmHg after 4 weeks and 0.06 mmHg after 6 weeks, while in the Ketogenic Diet: Low-Fat Diet group, they were −3.35 mmHg, −2.01 mmHg and −3.18 mmHg, and in the Ketogenic Supplement: Low-Fat Diet group −6.66 mmHg, −4.32 mmHg and −3.24 mmHg. The average weight loss was 6 kg, 8 kg, and 7 kg in the KD + KS, KD + PL, and LFD groups, respectively (*p* < 0.05). Aldosterone levels increased by 88% and 144% in the KD + PL and KD + KS groups, but did not change in the LFD group after 6 weeks. Despite the increase, aldosterone was not correlated with cardiovascular parameters such as blood pressure and ejection fraction. A highly interesting RCT published in 2023 [67] compared the effects of the very low-carbohydrate (VLC, ketogenic) diet against the DASH diet (with or without behavioural support) on blood pressure, blood glucose levels, and body weight in people with hypertension, prediabetes or type 2 diabetes, and overweight or obesity. It was shown that compared to the DASH diet, the VLC diet led to a significantly greater reduction in mean systolic blood pressure (−9.77 mm Hg vs. −5.18 mm Hg; *p* = 0.046), a decrease in glycated haemoglobin (−0.35% vs. −0.14%; *p* = 0.034), and a reduction in body weight (−19.14 pounds vs. −10.34 pounds; *p* = 0.0003). Importantly, additional behavioural support had no statistically significant effect on any of the parameters analysed. These results are particularly interesting in light of the fact that the DASH diet was designed specifically to lower blood pressure, yet it was inferior to the ketogenic diet in this regard. Another RCT assessed whether the low-calorie ketogenic diet (LCKD) with continuous positive airway pressure (CPAP) before bariatric surgery (BS) controlled the obstructive sleep apnoea syndrome (OSAS) more efficiently than CPAP alone [186]. It was found that LCKD + CPAP reduced the mean systolic (from 142.8 ± 13.3 mmHg to 133 ± 11.9 mmHg) and diastolic blood pressure (from 85.4 ± 8.38 mmHg to 78.7 ± 6.43 mmHg) to a greater extent than CPAP alone (decrease in SBP from 134.2 ± 10.4 mmHg to 130 ± 9.7 mmHg and DBP from 87 ± 11.6 mmHg to 82 ± 9.5 mmHg) in patients with severe obstructive sleep apnoea syndrome. Additionally, the combination of LCKD + CPAP was more effective in reducing CRP (on average from 6.12 ± 5.96 to 2.66 ± 2.57 vs. from 5.95 ± 5.9 to 6.36 ± 6.0 in the CPAP group), body weight (on average from 143.6 ± 23.6 to 129.7 ± 23.7 kg vs. from 132.7 ± 23 to 131.6 ± 22.3 kg in the CPAP group), total cholesterol (on average from 200.1 ± 30.1 to 180.4 ± 35.2 mg/dL vs. from 196.1 ± 32.9 to 180.8 ± 33.0 mg/dL in the CPAP group), LDL (on average from 127.4 ± 26.8 to 107.1 ± 37.1 mg/dL vs. from 128 ± 30.2 to 112.9 ± 34.9 mg/dL in the CPAP group), and triglycerides (on average from 191 ± 41.7 to 130 ± 79 mg/dL vs. from 151.6 ± 62.5 to 129.7 ± 62.2 mg/dL in the CPAP group). Another RCT [187] examined the effect of the ketogenic diet against a non-ketogenic diet in women doing strength training. The authors reported that women following the KD had a greater reduction in systolic blood pressure compared to the control group (on average −6.3 ± 6.0 [−10.5, −2.0] mmHg vs. −0.4 ± 8.9 [−6.8, 6.0] mmHg) and increased bone mineral density (BMD) (by an average of 0.02 ± 0.02 [0.01, 0.03] g/cm^2^ vs. 0.00 ± 0.02 [−0.02, 0.02] g/cm^2^). The authors of another interesting RCT [188] chose to compare three types of ketogenic diets: whey protein group [WPG], vegetable protein group [VPG], and animal protein group [APG] with very low calorie intake (VLCKD). The diets were compared in terms of their effectiveness, safety, and impact on the gut microbiota in people with insulin resistance and obesity. One of the parameters studied was blood pressure, which decreased in each group. The average SBP changes were as follows: from 132 ± 10 mmHg to 124 ± 13 mmHg in the WPG group; from 131 ± 8 mmHg to 121 ± 10 mmHg in the VPG group; and from 129 ± 9 mmHg to 121 ± 16 mmHg in the APG group. In turn, DBP decreased from 78 ± 11 mmHg to 70 ± 9 mmHg in the WPG group, from 78 ± 10 mmHg to 72 ± 10 mmHg in the VPG group, and from 78 ± 10 mmHg to 71 ± 9 mmHg in the APG group. In addition, all patients experienced a reduction in body weight, blood pressure, waist circumference, HOMA index, insulin, and total and LDL cholesterol levels.

A review of older (≥10 years) RCT studies suggests that ketogenic diets (KDs) promote blood pressure reduction, although in some cases, the effect is similar to that in control groups. One 52-week RCT [189] compared a very low-carbohydrate (LC) diet with a high-carbohydrate, low-fat (HC) diet in T2D patients. Blood pressure reduction was observed in both groups: LC −7.1 (−10.6; −3.7) mmHg SBP and −6.2 (−8.2; −4.1) mmHg DBP; HC −5.8 (−9.4; −2.2) mmHg SBP and −6.4 (−8.4; −4.3) mmHg DBP. While both diets also led to weight loss and lower HbA1c and fasting blood glucose levels, the LC diet was associated with greater improvement in the lipid profile, more stable blood glucose and reduced need for antidiabetic medication. Another interesting RCT by Yancy et al. [190] compared the effects of the low-carbohydrate ketogenic diet (LCKD) without calorie restriction with a low-fat, calorie-restricted diet supplemented with orlistat (O + LFD). Despite unrestricted calorie intake in the LCKD group, body weight loss was comparable to that in the O + LFD group (−9.5% e vs. −8.5%). Furthermore, the LCKD diet had a more beneficial effect on blood pressure: systolic blood pressure decreased by −5.9 vs. 1.5 mmHg, and diastolic blood pressure decreased by −4.5 vs. 0.4 mmHg compared to O + LFD, with both groups experiencing similar improvements in HDL cholesterol and triglyceride levels. Yet another RCT [191] was designed to compare changes in body weight and other cardiovascular risk factors in three energy-restricted diets (VLC-very low carbohydrate, VLF-very low fat and HUF-high unsaturated fat). After 15 months, blood pressure decreased significantly in each group: VLC −10.6 (10.6) mmHg SBP and −6.6 (12.1) mmHg DBP; VLF −6.0 (13.3) mmHg SBP and −7.5 (8.7) mmHg DBP; HUF −5.4 (13.3) mmHg SBP and −9.0 (9.3) mmHg DBP. In the control group, the results were as follows: 1.9 (8.3) mmHg SBP and 2.9 (8.2) mmHg DBP. Significant body weight loss was also reported in each group (except for the control group). Another RCT [192] compared a low-energy, very low-carbohydrate, high-saturated fat (LC) diet against an isocaloric, high-carbohydrate, low-fat (LF) diet in terms of their effect on endothelial function after 12 months in overweight and obese patients. After 12 months, blood pressure was significantly lower in both groups (LC −14 ± 2/−6 ± 2 mmHg, LF −15 ± 3/−8 ± 2 mmHg), as was body weight (LC −14.9 ± 2.1 kg, LF −11.5 ±1.5 kg) and pulse wave velocity (PWV), while flow-mediated dilatation (FMD) decreased in LC but remained unchanged in LF. Another RCT [193] also demonstrated that LC and LF diets promoted similar reductions in blood pressure: in the LC group, systolic pressure decreased from 132.7 ± 2.3 mmHg to 118.9 ± 2.0 mmHg and diastolic pressure from 72.3 ± 1.8 mmHg to 66.0 ± 2.0 mmHg; in the LF group, SBP decreased from 135.2 ± 2.1 mmHg to 120.6 ± 2.9 mmHg and DBP decreased from 77.1 ± 1.8 mmHg to 69.2 ± 1.7 mmHg over 52 weeks. Furthermore, similar improvements in body weight, glucose, insulin, insulin resistance, and CRP were observed after 12 months. These findings are consistent with those of another RCT [194], which reported that blood pressure, body weight, CRP, fasting glucose, and insulin concentrations decreased to a similar extent and were accompanied by weight loss in both diets (very-low-carbohydrate, high-fat (VLCHF) diet and a high-carbohydrate, low-fat (HCLF) diet). In the VLCHF group, SBP decreased from 133.1 ± 14.4 mmHg to 120.8 ± 11.5 mmHg and DBP from 73.6 ± 11.6 mmHg to 69.0 ± 11.7 mmHg after 24 weeks; in turn, in the HCLF group, SBP decreased from 136.1 ± 12.6 mmHg to 125.2 ± 15.8 mmHg and DBP from 77.8 ± 10.1 mmHg to 72.3 ± 9.01 mmHg. A 2003 study (the earliest of all RCTs discussed) [195] compared a very low-carbohydrate diet used ad libitum against a calorie-restricted diet (30% of energy from fat) in terms of their effect on body composition and cardiovascular risk factors in obese women considered healthy at the time. Blood pressure did not change significantly, as it was normal at the beginning of the study, after 3 months, and after 6 months. In the low-carbohydrate group, the BP values were initially 116/79 (3.23/2.69) mmHg, and changed to 112/72 (2.36/2.06) mmHg after 3 months, and to 114/74 (2.82/2.23) mmHg after 6 months. In the low-fat group, the BP values were 115/75 (2.47/1.99) mmHg at the beginning, 116/75 (2.01/1.79) mmHg after 3 months, and 113/74 (2.41/1.62) mmHg after 6 months. Significant differences were observed in terms of body weight and fat loss: women in the low-carbohydrate group, despite no calorie restrictions, lost more weight (8.5 ± 1.0 kg vs. 3.9 ± 1.0 kg) and fat (4.8 ± 0.67 kg vs. 2.0 ± 0.75 kg) compared to the low-fat diet group. This study also confirms that the calorie deficit in low-carbohydrate diets is achieved intuitively, without any formally required energy intake restriction.

It should also be noted that adherence support varied across the included studies. While some interventions incorporated structured dietary counselling and regular follow-up, others, such as the ad libitum ketogenic intervention by Brehm et al. [195], allowed participants to eat until satiety without prescribed caloric restriction. Despite this, significant reductions in body weight and blood pressure were observed, supporting the hypothesis that the increased satiety associated with ketogenic diets may promote spontaneous energy restriction and facilitate long-term adherence.

A summary of the randomised controlled trials described in this section is given in Table 2.

## 8. Ketogenic Diet as a Factor Lowering and Raising Blood Pressure

As analysed in Section 5 and demonstrated in numerous clinical studies, the ketogenic diet can effectively reduce blood pressure. Importantly however, its impact is not always clear-cut and may depend on several key factors, including electrolyte supply and electrolyte levels.

Firstly, to enhance the hypotensive effect of the KD and prevent a potential increase in blood pressure, it is important to ensure adequate potassium intake and level. As described in Section 6.4.1, this micronutrient has a strong hypotensive effect, acting through a number of mechanisms. Meanwhile, its deficiency (hypokalaemia) leads to increased blood pressure, arrhythmia, palpitations, muscle cramps, weakness, fatigue, and constipation [196]. One study even showed an inverse relationship between blood potassium levels and blood pressure, confirming the close pathophysiological relationship between serum potassium concentration and primary HTN [197]. Another study confirmed that higher potassium intake alone is inversely correlated with changes in blood pressure as early as puberty [198]. In turn, potassium deficiency is associated with an increase in blood pressure. A non-linear dependency analysis showed that a total daily potassium intake of approximately 4500 mg in people without hypertension and 4500–6500 mg in people with hypertension was associated with the greatest blood pressure reduction. Therefore, both low and very high potassium intake (less common, mainly as a result of supplementation and in selected subgroups of patients) may be associated with increased blood pressure, as demonstrated in one meta-analysis [199]. At the same time, increased potassium intake is not safe for all patients, especially in the case of chronic kidney disease [200] and the use of ACE inhibitors, AT_1_ receptor antagonists, aldosterone antagonists or trimethoprim, which is associated with the risk of hyperkalaemia. Therefore, decisions regarding potassium intake should be tailored to the patient and based on laboratory test assessment. Meanwhile, as described in Section 6.4, potassium concentration after switching to the ketogenic diet may decrease due to increased diuresis, which is a purely physiological response of the body. It has been estimated that potassium (kaliuresis) and sodium (natriuresis) excretion is strongest between days 1 and 4 and subsides after approximately 14 days after switching to the KD, a time that coincides with most of the symptoms of a condition known as keto-flu, which may (but does not have to, if electrolyte intake and hydration are adequate) develop in the first days of KD [144]. After the accelerated excretion of potassium in the urine subsides, the question of its adequate supply in the diet still needs to be addressed, as it is crucial for maintaining the correct concentration of this electrolyte in the blood. While some authors report that people on low-carbohydrate diets rarely consume enough potassium, the problem actually affects the general population. Therefore, as with any other dietary model, an adequate supply of potassium must be ensured [201].

Secondly, it is important to ensure an adequate supply of magnesium. The increased diuresis in the early stages of the ketogenic diet promotes increased magnesium excretion and thus increases the risk of hypomagnesaemia, as confirmed, for instance, in patients taking certain diuretic drugs [202,203]. As with potassium, once the increased urinary excretion subsides, the issue of adequate long-term dietary intake remains. It has been suggested that people on low-carbohydrate diets may not be consuming enough magnesium, but again, this is a problem that affects the general population [201]. The ketogenic diet is therefore no exception—as with other dietary models, care should be taken to ensure an adequate daily intake of magnesium, defined in official standards at 400 to 420 mg for men and 310 to 320 mg for women [204]. Interestingly, in one study of individuals on the Palaeolithic ketogenic diet, blood magnesium levels were normal in 49 out of 50 people, without any supplementation. Quite importantly, the authors observed an inverse correlation between glucose and magnesium levels and suggested that the high prevalence of magnesium deficiency (previously associated with various chronic diseases) is correlated with a carbohydrate-based diet (typical of Western culture) and not with the chronic disease itself [205].

Thirdly, proper hydration is essential. In the early stages of the ketogenic diet, increased diuresis causes increased body water loss, which largely explains the rapid initial weight loss [19]. With less water retained by the body, plasma volume may decrease, and consequently, blood pressure may drop—hence the effectiveness of diuretic drugs in the treatment of hypertension [206]. On the other hand, however, prolonged insufficient fluid intake can have the opposite effect, increasing blood pressure due to compensatory mechanisms associated with fluid loss and vasoconstriction. It has been demonstrated that hypohydration (a state of reduced water content in the body) can impair the endothelial function, activate the RAA and vasopressin systems, increase sympathetic activity, raise the endothelin-1 concentration and cause arterial stiffness as well as impaired orthostatic tolerance [207]. Therefore, adequate hydration throughout the ketogenic diet is essential, as in other dietary plans that also require adequate fluid intake. However, particular attention should be paid to this issue in the initial phase of the ketogenic diet, as diuresis may cause the body to require even more water than under normal conditions.

Fourthly, blood pressure increase in people following the ketogenic diet may be due to the metabolic stress that accompanies the process of keto-adaptation. In this initial period, the body must adapt to using ketone bodies (instead of glucose) as its main source of energy. This transition, although beneficial in the long-term, is challenging for the metabolic system and may cause symptoms collectively known as “keto flu” [26,144,208,209]. While these symptoms, associated with the loss of electrolytes and water, can most likely be alleviated by adequate hydration and electrolyte supplementation [144], the adaptation process itself is nevertheless stressful for the body, which can cause additional mental stress. Keto flu can temporarily increase the cortisol and adrenaline levels and the activity of the sympathetic nervous system, which prepares the body for “fight or flight” situations [210,211,212,213]. Hence, these changes offer a physiological explanation for a possible short-term increase in blood pressure, especially in the initial phase of the ketogenic diet. However, it appears that the hypotensive mechanisms associated with the switching to a KD significantly outweigh the hypertensive ones, although each body’s response may be different. Even if the blood pressure increases temporarily in response to the mechanisms outlined above, once the keto-adaptation process is complete (i.e., when the body adapts to the new energy source and electrolyte and hydration levels are properly regulated), blood pressure should stabilise and return to normal in healthy individuals or decrease in those with hypertension. In addition, transient symptoms accompanying keto-adaptation, such as headache, fatigue, or lethargy, may contribute to early discontinuation of the diet in some individuals. This should be taken into account when interpreting the results of clinical trials, particularly intention-to-treat analyses.

**Figure 4:**
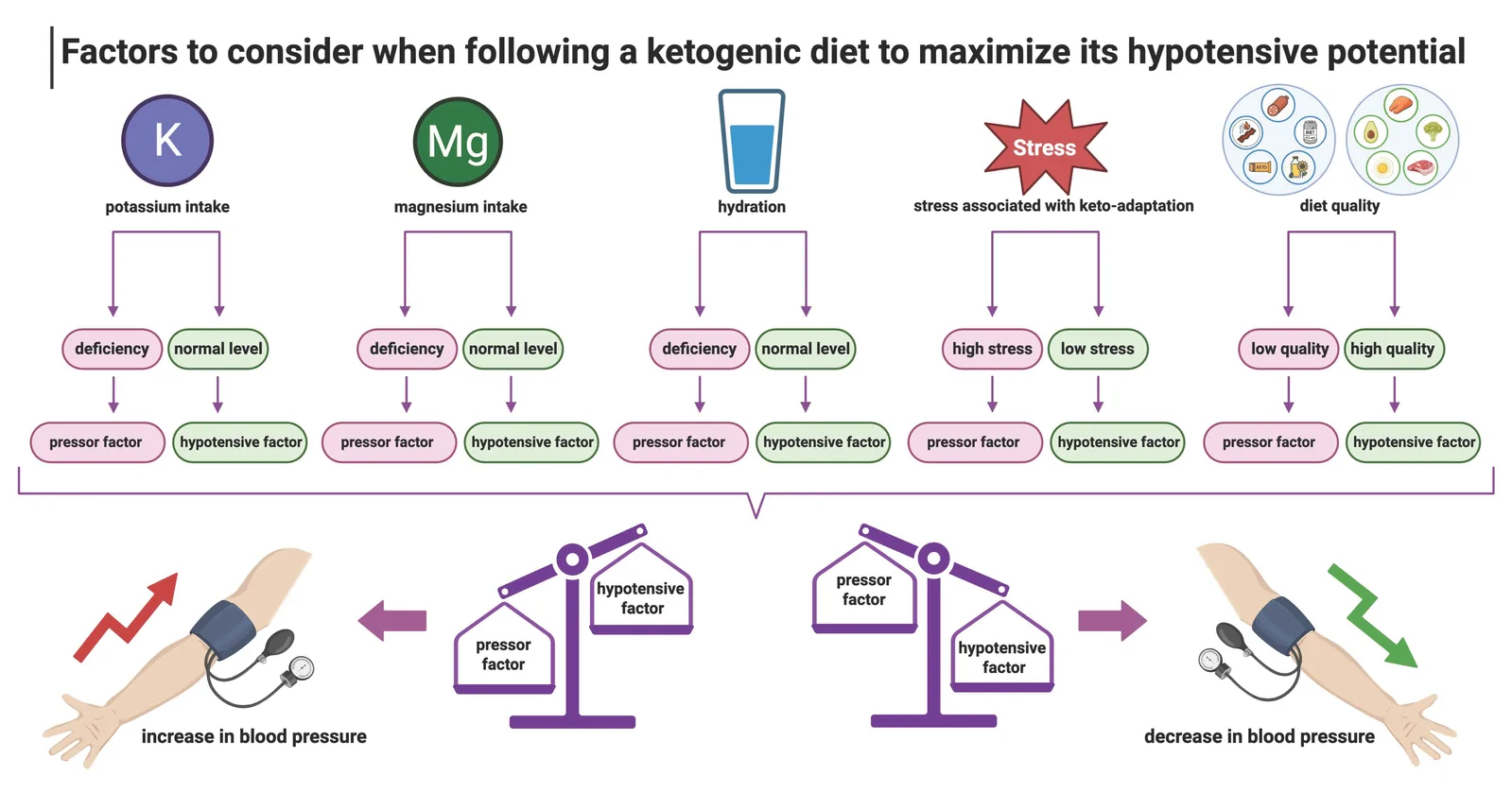
Factors to consider when following the ketogenic diet to maximise its hypotensive potential. Created in BioRender. https://BioRender.com/85vghr9 (accessed on 30 June 2026).

Fifthly, attention must be paid to the quality of the ketogenic diet, which should be based on natural, minimally processed products [36]. Poor-quality products marketed as “keto”, including highly processed meal replacements, low-carb snacks, diet drinks or ready-made keto meals, combined with a lack of concern for wholesome products, can trigger many adverse nutritional factors simultaneously. Although the KD eliminates sugars and significantly reduces carbohydrate intake, it does not eliminate many other adverse nutritional factors, such as trans fatty acids, food additives, or excess omega-6 fatty acids. As in any other diet based on low-quality and highly processed foods, these substances will have a negative impact on metabolic and cardiovascular health [214,215,216,217,218]. All the negative effects associated with consuming low-quality processed foods, combined with a deficiency of nutrients, can adversely affect blood pressure. Therefore, even a ketogenic diet that meets the quantitative criteria for macronutrients but is based on low-quality products may lose its health-promoting potential, and in some cases, even cause some additional cardiovascular risks. It is therefore important to ensure an adequate quality of the products consumed by people on a KD. Figure 4 summarises the factors that should be considered to maximise the diet’s hypotensive potential.

Importantly, in pharmacologically treated hypertensive patients, the ketogenic diet requires special attention, particularly during the first weeks of implementation, when rapid reductions in blood pressure and increased diuresis may necessitate close clinical monitoring and timely adjustment (usually reduction) of antihypertensive medication dosages, especially in patients receiving diuretics or ACE inhibitors [219].

## 9. Strengths and Limitations

This narrative review is a comprehensive summary of the available literature, including meta-analyses, systematic reviews, randomised controlled trials, clinical trials, case reports, mechanistic evidence, and current recommendations. The search strategy followed by the authors made it possible to identify studies evaluating both the effect of the ketogenic diet on blood pressure in humans and the potential mechanisms underlying that effect. An additional advantage of the review is that it takes into account the quality of the ketogenic diet itself and indicates the possible differences between its variants in terms of clinical outcomes. Last but not least, the review identifies factors that may maximise the diet’s hypotensive effect.

The review has a narrative rather than systematic design, and therefore does not include a predefined study selection protocol, formal quality assessment, or systematic evidence synthesis. These are inherent limitations of the narrative review approach and may increase the risk of selection bias. Nevertheless, the narrative design was considered appropriate for the aim of this manuscript, namely to provide a comprehensive overview of the available evidence regarding the relationship between the ketogenic diet and blood pressure, including both mechanistic and clinical aspects. The available studies are fairly heterogenous, which results from the differences between ketogenic diet versions, populations, and observation times. In many studies, the sample size was small and the intervention period was short, and blood pressure was often not the primary endpoint. Furthermore, the number of studies specifically looking at the long-term effects of the ketogenic diet on blood pressure and cardiovascular risk remains limited. Moreover, long-term randomised evidence evaluating the effects of dietary interventions on cardiovascular events, cardiovascular mortality, all-cause mortality, and long-term adherence remains limited across virtually all dietary models, including the ketogenic diet. Therefore, further well-designed prospective studies with extended follow-up are needed to determine whether the short-term improvements observed with different dietary interventions translate into long-term clinical benefits.

## 10. Conclusions

A number of key conclusions come to mind:

1. Abdominal obesity is strongly correlated with hypertension (HTN). Since the KD increases satiety, reduces appetite, offers rapid initial weight loss, improves glycaemic and insulinemic responses, and promotes intuitive calorie deficit (in addition to a few other advantages), it may be more effective than other dietary strategies in promoting weight loss in many individuals, which is an important factor in lowering blood pressure in patients with HTN coexisting with overweight or obesity.
2. Insulin resistance, common in patients with HTN, plays an important role in its pathogenesis. Importantly, the condition may also affect slim people with a normal BMI, in whom insulin resistance significantly increases the risk of developing hypertension, despite normal body weight. The KD can be helpful in managing insulin resistance thanks to, among other things, effective weight loss (in overweight or obese individuals), low glycaemic index (GI), greater glycaemic and insulin stability, reduced inflammation, and a beneficial effect on insulin signalling pathways, all of which improve tissue sensitivity to this hormone.
3. Although BMI remains widely used in clinical research as a measure of obesity—including in many of the studies reviewed in this paper—it is a low-sensitivity indicator with well-documented limitations. It fails to distinguish between fat and lean mass, does not reflect fat distribution, and may misclassify both metabolically obese individuals of normal weight and metabolically healthy individuals with excess weight. In the context of hypertension, where visceral adiposity appears to be a more relevant pathophysiological driver than overall body weight, BMI should not be used as the sole diagnostic criterion for obesity. Direct measures of body composition and fat distribution—such as waist circumference, waist-to-height ratio, or imaging-based assessment of visceral adipose tissue—are more clinically informative and should be prioritised where possible.
4. There is a particularly strong association between visceral adipose tissue (much stronger than between subcutaneous fat, for example) and hypertension and the risk of its development. The KD effectively reduces that tissue, even in people of normal weight. Some of the reviewed studies suggest that it may outperform other dietary models in this regard, making it an important tool in HTN prevention and treatment.
5. Water and electrolyte balance is another important element in blood pressure regulation, and the ketogenic diet, especially in its initial stage, has a significant effect in this regard. By rapidly depleting glycogen stores and lowering insulin levels, the diet increases diuresis and sodium, potassium, and magnesium loss, thus reducing plasma volume and potentially lowering blood pressure. This phenomenon is most pronounced during keto-adaptation, when lower insulin levels limit sodium reabsorption in the kidneys and reduce the activity of the renin–angiotensin–aldosterone system (RAA). At this stage, it is particularly important to replenish potassium and magnesium in order to maintain the beneficial hypotensive effect of the diet.
6. Inflammation plays an important role in the development and progression of HTN, and the KD significantly reduces it, thus promoting blood pressure reduction. A key mechanism in this regard is the inhibition of NLRP3 inflammasome activity by β-hydroxybutyrate (BHB), which reduces the secretion of pro-inflammatory cytokines and supports blood pressure regulation. In addition, KD meals contain minimum quantities of pro-inflammatory simple sugars (below the values allowed by standard dietary recommendations) and have a very low GI (also lower than standard recommendations), which improves glycaemic and insulinemic responses. All of these factors help lower blood pressure.
7. Most meta-analyses, systematic reviews, and randomised controlled trials in humans demonstrate that the KD effectively lowers blood pressure, although this effect is not always statistically significant and does not always exceed that of comparable diets.
8. It is plausible that the reduction in blood pressure associated with the ketogenic diet may be greater in patients with hypertension than in individuals with normal baseline blood pressure.
9. To maximise the hypotensive effect of KD, adequate potassium and magnesium intake, proper hydration, reduction in additional stress factors during the adaptation period, and high-quality foods should be ensured. If these aspects are not properly managed, blood pressure may increase or remain unchanged, thus offsetting the diet’s beneficial effect on blood pressure. It should also be emphasised that during the initial phase of adaptation to a ketogenic diet, there may be an increased requirement for sodium, partly due to enhanced natriuresis induced by carbohydrate restriction. Consequently, paradoxically, these patients may require a higher dietary sodium intake than prior to the implementation of the nutritional intervention.
10. There is a clear need for more high-quality studies investigating the effect of the ketogenic diet on systolic and diastolic blood pressure as the main endpoint.
11. Future studies should place particular emphasis on assessing the qualitative composition of ketogenic diets and clearly determining the extent to which the observed hypotensive effect is due to weight loss alone and to other factors.
12. People taking antihypertensive medication should exercise particular caution before switching to the ketogenic diet. In consultation with their doctor, they should work out a personalised plan for modifying or reducing the drug dosage in order to minimise the risk of hypotension. In this patient population, regular monitoring of blood pressure is essential, with particular attention to periods of elevated ambient temperature, which may further exacerbate reductions in blood pressure.

## Figures and Tables

**Table 1:** Ketogenic diet and blood pressure and other cardiometabolic parameters—findings from meta-analyses and systematic reviews.

Year of Study, References	Study Aim	Groups	Effect on Blood Pressure and Other Parameters
2024 [175]	To assess the effect of a very low-calorie ketogenic diet (VLCKD) on cardiovascular risk factors in patients with type 2 diabetes	29 clinical trials involving 2359 participants group 1 = very low-calorie ketogenic diet (VLCKD) group 2 = control diets	Results vs. control diets: -reduced systolic blood pressure (WMD = −2.85 mmHg), -reduced diastolic blood pressure (WMD = −1.40 mmHg), -reduced systolic blood pressure in the subgroup of individuals with a BMI > 35 kg/m^2^ (WMD = −3.15 mmHg) -reduced levels of fasting glucose (WMD = −11.68 mg/dL), HbA1c (WMD = −0.29), insulin (WMD = −1.45), HOMA-IR (WMD = −0.71) and triglycerides (WMD = −17.95) -all of the above results were statistically significant
2024 [176]	To assess the impact of ketogenic diets on cardiovascular risk factors in RCT studies	27 RCTs involving 1278 participants group 1 = ketogenic diets group 2 = control diets	Results vs. control diets: -reduced diastolic blood pressure (WMD = −1.41 mmHg), -no significant SBP differences -lower levels of triglycerides (WMD = −0.20 mmol/L), glucose (WMD = −0.18 mmol/L), insulin (WMD = −8.32 pmol/L), body weight (WMD = −2.59 kg) and BMI (WMD = −1.59 kg/m^2^) -higher levels of HDL (WMD = 0.16 mmol/L), LDL (WMD = 0.35 mmol/L) and total cholesterol (WMD = +0.36 mmol/L)
2024 [177]	To examine the effect of ketogenic diets on blood pressure based on the available literature	23 RCTs involving 1664 participants group 1 = ketogenic diets group 2 = control diets	Results vs. control diets: -reduced systolic blood pressure (WMD = −0.87 mmHg), statistically insignificant -reduced diastolic blood pressure (WMD = −0.11 mmHg), statistically insignificant -no association between the percentage of calories from fat in the KD diet and blood pressure levels
2022 [178]	To assess the dose-dependent effect of carbohydrate restriction in patients with type 2 diabetes	50 clinical trials involving 4291 participants Group 1: High carbohydrate (>45%) diets Group 2: Low carbohydrate (≤45%) diets	Compared to a diet with 65% of energy coming from carbohydrates, a 10% reduction in carbohydrate intake was associated with a mean gradual decrease in SBP: for 55% −2.37 mmHg (−6.99 to −2.34), 45% −4.59 mmHg (−8.21 to −0.97), 40% −5.44 mmHg (−9.41 to −1.47), 35% −6.17 mmHg (−9.87 to −2.12), 30% −6.86 mmHg (−10.80 to −3.12), 25% −7.56 mmHg (−10.98 to −3.29), 20% −8.25 mmHg (−12.0 to −4.25), 15% −8.95 mmHg (−12.25 to −4.50), and 10% −9.64 mmHg (−12.6 to −6.7).
2022 [179]	To assess the level of scientific evidence on the benefits and harms of carbohydrate-restricted diets and intermittent fasting in order to propose reasonable recommendations	50 RCTs (+8 RCTs looking at IF) Group 1: carbohydrate-restricted diets (divided by carbohydrate content) Group 2: control diets (mainly low-fat (44.7%) and calorie-restricted (29.8%) diets) Group 3: intermittent fasting	-reduced mean systolic blood pressure in VLCD by −4.97 mmHg vs. in control groups by an average of −3.0 mmHg (mean difference: −1.97 (−3.68 to −0.25) for up to 6 months and by 8.1 mmHg (95% CI, −13.35 to −2.85) over periods exceeding 6 months (up to 1 year) -no significant differences in diastolic blood pressure reduction: −2.78 mmHg vs. −2.1 mmHg in control groups (mean difference: −0.68 (−1.79 to 0.44)) in overweight or obese individuals - no significant differences in blood pressure changes in adults with type 2 diabetes (−1.36 mmHg SBP and −1.12 mmHg DBP in VLCD vs. 1.7 mmHg and −2.5 mmHg in control groups) -VLCD groups reported greater reductions in body weight (−7.42 kg vs. −3.75 kg in the control group), fat mass (−7.81 kg vs. −4.8 kg) and waist circumference (−8.81 cm vs. −4.7 cm), as well as a reduction in BMI (−2.88 vs. −1.0), fat-free mass (−1.35 kg vs. −0.3 kg), HbA1c (−0.42 vs. −0.15), fasting insulin (−2.92 μU/mL vs. −1.55 μU/mL), both in overweight/obese individuals, as well as in individuals with type 2 diabetes (HbA1C −0.56 vs. −0.2; HOMA-IR −1.52 vs. −0.45; fasting glucose −26.84 mg/dL vs. −17.2 mg/dL; body weight −7.24 kg vs. −3.4 kg). In both groups of patients, VLCD resulted in a greater reduction in TG and an increase in HDL and LDL.
2020 [180]	To assess the efficacy and safety of the very low-calorie ketogenic diet (VLCKD) in overweight and obese patients	12 clinical trials involving 801 participants (including 4 trials looking at blood pressure, involving 199 participants) group 1 = very low-calorie ketogenic diet (VLCKD) group 2 = depending on the study analysed, either none, or very-low-calorie diet (VLCD), or low-calorie diet (LCD)	-reduced systolic blood pressure (−8.5 mmHg (−11.4 to −5.6)) -reduced diastolic blood pressure (−7.2 mmHg (−8.9 to −5.5)) -reduced body weight (average −10 kg up to 4 weeks and −15.6 kg > 4 weeks KD) and BMI (−5.3 kg/m^2^), waist circumference (−12.6 cm), HbA1c concentration (−0.7%), total cholesterol (−28 mg/dL), triglycerides (−30 mg/dL), AST (−7 U/L), ALT (−8 U/L) and GGT (−8 U/L)
2013 [181]	To assess whether VLCKD (≤50 g carbohydrates/day) has better long-term effects on weight loss and cardiovascular risk factors compared to a conventional low-fat, calorie-restricted diet (LFD, <30% energy from fat).	13 clinical trials involving 1577 participants group 1 = very-low-carbohydrate ketogenic diet (VLCKD) group 2 = low-fat calorie-deficient diet (LFD)	Results vs. LFD diets: -reduced diastolic blood pressure (WMD = −1.43 mmHg), 95% CI: −2.49 to −0.37) -reduced systolic blood pressure (WMD = −1.47 mmHg); 95% CI: −3.44 to 0.50); no significant difference between the groups -body weight loss (WMD = −0.91 kg), lower triglyceride levels (WMD = −0.18 mmol/L) and higher HDL (WMD = +0.09 mmol/L) and LDL (WMD = +0.12 mmol/L) levels

VLCKD—Very Low-Calorie Ketogenic Diet; KD—Ketogenic Diet; WMD—Weighted Mean Difference; BMI—Body Mass Index; HbA1c—Haemoglobin A1c; HOMA-IR—Homeostasis Model Assessment of Insulin Resistance; RCT—Randomised Controlled Trial; SBP—Systolic Blood Pressure; DBP—Diastolic Blood Pressure; HDL—High-Density Lipoprotein; LDL—Low-Density Lipoprotein; IF—Intermittent Fasting; LCD—Low-Calorie Diet; LFD—Low-Fat Diet; AST—Aspartate Aminotransferase; ALT—Alanine Aminotransferase; GGT—Gamma-Glutamyl Transferase.

**Table 2:** Ketogenic diet and blood pressure and other cardiometabolic parameters—findings from randomised controlled trials (RCTs).

Year of Study, References	Study Aim	Groups	Effect on Blood Pressure and Other Parameters
2024 [182]	To assess the effectiveness of the newly developed Healthy Ketogenic Diet (HKD) compared to the energy-restricted diet (ERD) in terms of weight loss and improvement of metabolic parameters in obese adults.	Multiethnic Asian adults (*n* = 80) with a body mass index ≥ 27.5 kg/m^2^ group 1 = Healthy Ketogenic Diet (HKD) group 2 = energy-restricted diet (ERD)	HDK vs. ERD performance: -reduced systolic blood pressure (−7.7 ± 8.9 mmHg vs. −2.6 ± 12.2 mmHg in the ERD group; *p* = 0.005) -insignificantly reduced diastolic blood pressure (−3.7 ± 6.5 mmHg vs. −2.4 ± 7.7 mmHg in the ERD group) -body weight loss (−7.8 ± 5.2 kg vs. −4.2 ± 5.6 kg in the ERD group; *p* = 0.01) -lower HbA1c (−0.3 ± 0.3% vs. −0.1 ± 0.2% in the ERD group; *p* = 0.008) -reduced AST activity (−7.6 ± 15.5 IU/L vs. +0.6 ± 11.5 IU/L in the ERD group; *p* = 0.01) -no increase in LDL in either group
2024 [183]	To compare the Asian ketogenic diet (AKD, a diet with a balanced intake of protein and fat from Asian foods) against the balanced low-calorie diet (BLC) in individuals diagnosed with metabolic syndrome	group 1 = Asian ketogenic diet based on egg yolk (Yolk-AKD, *n* = 28) group 2 = Asian ketogenic diet based on egg white (White-AKD, *n* = 26) group 3 = balanced low-calorie diet (BLC)	AKD vs. BLC performance: -greater reduction in systolic and diastolic blood pressure: White-AKD = week 35: SBP 121.9 ± 2.2 mmHg; DBP 81.5 ± 1.4 mmHg, week 52: SBP 123.2 ± 1.9 mmHg; DBP 82.3 ± 1.2 mmHg, Yolk-AKD = week 35: SBP 126.3 ± 2.1 mmHg; DBP 87.2 ± 1.4 mmHg, week 52: SBP 127.7 ± 1.8 mmHg; DBP 87.6 ± 1.1 mmHg, BLC, week 35: SBP 132.2 ± 2.4 mmHg; DBP 88.7 ± 1.5 mmHg; week 52: SBP 134.2 ± 2.1 mmHg; DBP 91.7 ± 1.3 mmHg; -Between weeks 35 and 52, the AKD group showed reduced anthropometric parameters, improved glucose tolerance, improved lipid profiles, and improved liver function compared to the BLC group.
2024 [184]	To assess whether the ketogenic diet, with or without ketone ester supplementation, has a beneficial effect on insulin sensitivity (liver, muscle and adipose tissue) and selected metabolic parameters, compared to a standard diet, in the absence of weight loss.	29 overweight and obese individuals group 1 = ketogenic diet maintaining body weight group 2 = ketogenic diet maintaining body weight with beta-hydroxybutyrate (β-OH-B) ketone ester supplementation group 3 = standard weight-maintaining diet	-no significant changes in blood pressure in the ketogenic diet groups: -regular ketogenic diet: SBP changed from 125 ± 3 to 125 ± 2 mmHg (*p* = 0.867) and DBP from 78 ± 3 to 77 ± 3 mmHg (*p* = 0.773); -ketogenic diet with ketone ester supplementation: SBP changed from 124 ± 2 to 121 ± 3 mmHg (*p* = 0.463) and DBP from 76 ± 2 to 76 ± 2 mmHg *p* = 0.976 -standard diet: SBP changed from 134 ± 2 mmHg to 123 ± 4 mmHg (*p* = 0.048) and DBP from 80 ± 3 to 73 ± 3 mmHg (*p* = 0.158). -in all 3 groups, no significant changes in plasma lipid profile and insulin sensitivity of the whole body (muscles), liver and adipose tissue
2023 [185]	To investigate effect of the ketogenic diet (KD) with varying sodium content versus that of the low-fat diet (LFD) on the RAA system in overweight and obese adults	28 participants group 1 = ketogenic diet + ketone salt supplementation (KD + KS) group 2 = ketogenic diet + placebo (KD + PL) group 3 = low-fat diet (LFD) post hoc	-no significant changes in blood pressure between the groups: -Differences in SBP (mmHg) between the Ketogenic Diet: Ketogenic Supplement group were −0.54 (−8.62, 7.54) in week 2, 2.62 (−5.46, 10.70) in week 4, and −6.87 (−14.95, 1.21) in week 6; in the Ketogenic Diet: Low-Fat Diet group, they were −6.34 (−14.43, 1.75), −4.51 (−12.60, 3.58) and −6.21 (−14.30, 1.88), respectively; and in the Ketogenic Supplement: Low-Fat Diet group −5.80 (−13.97, 2.36), −7.13 (−15.30, 1.03) and 0.66 (−7.51, 8.82); -Differences in DBP (mmHg) in the Ketogenic Diet: Ketogenic Supplement group: 3.31 (−3.14, 9.76) (week 2), 2.31 (−4.14, 8.76) (week 4) and 0.06 (−6.39, 6.52) (week 6); in the Ketogenic Diet: Low-Fat Diet group, respectively: −3.35 (−9.81, 3.11), −2.01 (−8.47, 4.45) and −3.18 (−9.64, 3.28); and in the Ketogenic Supplement: Low-Fat Diet group: −6.66 (−13.16, −0.15), −4.32 (−10.83, 2.18) and −3.24 (−9.74, 3.26) -weight loss after 6 weeks by an average of 6, 8 and 7 kg in the KD + KS, KD + PL and LFD groups, respectively (*p* < 0.05) -aldosterone levels increased by 88% and 144% in the KD + PL and KD + KS groups, respectively, with no changes in the LFD group after 6 weeks - decreased renin levels in all groups -No correlation between aldosterone and cardiovascular parameters (blood pressure and ejection fraction)
2023 [67]	To compare the effects of a very low-carbohydrate (VLC) diet (a ketogenic diet) and DASH diet, with or without behavioural support, on blood pressure, blood glucose levels and body weight in individuals with hypertension, prediabetes or type 2 diabetes and overweight or obesity	94 participants group 1 = very low-carbohydrate (VLC) (ketogenic diet) group 2 = DASH diet	VLC vs. DASH performance: -reduced mean systolic blood pressure (−9.77 mmHg vs. −5.18 mmHg; *p* = 0.046) -reduced glycated haemoglobin (−0.35% vs. −0.14%; *p* = 0.034) -weight loss (−19.14 pounds vs. −10.34 pounds; *p* = 0.0003) -no data on DBP changes
2022 [186]	To assess whether the low-calorie ketogenic diet (LCKD) with continuous positive airway pressure (CPAP) before bariatric surgery (BS) controlled the obstructive sleep apnoea syndrome (OSAS) more efficiently than CPAP alone	70 participants group 1 = low-calorie ketogenic diet (LCKD) + continuous positive airway pressure (CPAP) group 2 = continuous positive airway pressure (CPAP)	LCKD + CPAP vs. CPAP performance: -reduced mean systolic blood pressure (from 142.8 ± 13.3 mmHg to 133 ± 11.9 mmHg) compared to CPAP alone (decrease in SBP from 134.2 ± 10.4 mmHg to 130 ± 9.7 mmHg -reduced mean diastolic blood pressure (from 85.4 ± 8.38 mmHg to 78.7 ± 6.43 mmHg) compared to CPAP alone (decrease in DBP from 87 ± 11.6 mmHg to 82 ± 9.5 mmHg) -reduced CRP (on average from 6.12 ± 5.96 to 2.66 ± 2.57 vs. from 5.95 ± 5.9 to 6.36 ± 6.0 in the CPAP group) -reduced body weight (on average from 143.6 ± 23.6 to 129.7 ± 23.7 kg vs. from 132.7 ± 23 to 131.6 ± 22.3 kg in the CPAP group) -reduced total cholesterol level (on average from 200.1 ± 30.1 to 180.4 ± 35.2 mg/dL vs. from 196.1 ± 32.9 to 180.8 ± 33.0 mg/dL in the CPAP group); LDL (on average from 127.4 ± 26.8 to 107.1 ± 37.1 mg/dL vs. from 128 ± 30.2 to 112.9 ± 34.9 mg/dL in the CPAP group) and triglycerides (mean from 191 ± 41.7 to 130 ± 79 mg/dL vs. from 151.6 ± 62.5 to 129.7 ± 62.2 mg/dL in the CPAP group)
2021 [187]	To assess the effect of the ketogenic diet on blood pressure, visceral adipose tissue (VAT), bone mineral content (BMC), and bone mineral density (BMD) in resistance-trained women.	21 participants (females) group 1 = ketogenic diet (KD) group 2 = non-ketogenic diet (NKD)	KD vs. NKD performance: -reduced systolic blood pressure (mean −6.3 ± 6.0 [−10.5, −2.0] mmHg vs. −0.4 ± 8.9 [−6.8, 6.0] mmHg in the NKD group) -increased bone mineral density (BMD) (mean 0.02 ± 0.02 [0.01, 0.03] g/cm^2^ vs. 0.00 ± 0.02 [−0.02, 0.02] g/cm^2^ in the NKD group) -no significant effect on VAT in either group
2020 [188]	To compare isocaloric VLCKD diets containing different protein sources (whey, plant, animal) with identical caloric content ≤800 kcal/day in terms of efficacy, safety and impact on gut flora composition in obese and insulin-resistant individuals over a 45-day period	48 participants group 1 = whey protein group [WPG] group 2 = vegetable protein group [VPG] group 3 = animal protein group [APG]	-mean SBP changes: from 132 ± 10 mmHg to 124 ± 13 mmHg in the WPG group; from 131 ± 8 mmHg to 121 ± 10 mmHg in the VPG group; from 129 ± 9 mmHg to 121 ± 16 mmHg in the APG group; -mean DBP changes: from 78 ± 11 mmHg to 70 ± 9 mmHg (WPG); from 78 ± 10 mmHg to 72 ± 10 mmHg (VPG); from 78 ± 10 mmHg to 71 ± 9 mmHg (APG). -reduced body weight, BMI, blood pressure, waist circumference, HOMA index, insulin and total cholesterol and LDL cholesterol concentrations in all groups
2015 [189]	To compare a very low-carbohydrate diet rich in unsaturated fats and low in saturated fats (LC) against a high-carbohydrate, low-fat diet (HC) in terms of 52-week effect on glycaemic control and selected cardiovascular risk factors in patients with T2D.	115 participants group 1 = very low-carbohydrate diet, rich in unsaturated fats and low in saturated fats (LC) group 2 = high-carbohydrate, low-fat diet (HC)	-reduced systolic blood pressure: LC −7.1 (−10.6; −3.7) mmHg vs. −5.8 (−9.4; −2.2) mmHg (HC) -reduced diastolic blood pressure: LC −6.2 (−8.2; −4.1) mmHg vs. −6.4 (−8.4; −4.3) mmHg (HC) -lower body weight, HbA1c and fasting blood glucose in both groups -in the LC group, greater improvement in lipid profile, glycaemic stabilisation and reduced need for antidiabetic medication
2010 [190]	To assess changes in body weight, metabolic parameters and adverse events over 48 weeks in outpatients randomly assigned to a low-carbohydrate ketogenic diet (LCKD) or orlistat therapy combined with a low-calorie, low-fat diet (O + LFD)	146 participants group 1 = low-carbohydrate, ketogenic diet (LCKD) group 2 = orlistat + low-fat diet (O + LFD)	LCKD had a more beneficial effect on blood pressure: -reduced systolic blood pressure (−5.9 vs. 1.5 mmHg in the O + LFD group), -reduced diastolic blood pressure (−4.5 vs. 0.4 mmHg in the O + LFD group), -similar improvement in HDL cholesterol and triglyceride levels in both groups
2010 [191]	To compare changes in body weight and other cardiovascular risk factors in three isocaloric energy-restricted diets against a control group without intervention after 1 year.	113 participants group 1 = VLC-very low carbohydrate group 2 = VLF-very low fat group 3 = HUF-high unsaturated fat group 4 = control (no intervention)	-significant reduction in blood pressure in each group compared to the control group: -VLC = −10.6 (10.6) mmHg SBP and −6.6 (12.1) mmHg DBP; VLF = −6.0 (13.3) mmHg SBP and −7.5 (8.7) mmHg DBP; HUF = −5.4 (13.3) mmHg SBP and −9.0 (9.3) mmHg DBP compared to the non-intervention group (1.9 (8.3) mmHg SBP and 2.9 (8.2) mmHg DBP) -body weight loss (−2.9 (4.9) kg in VLC; −2.1 (4.7) kg in VLF; −3.9 (6.3) kg in HUF vs. 0.8 (5.0) kg in the control group
2010 [192]	To compare a low-energy, very low-carbohydrate, high-saturated fat (LC) diet against an isocaloric, high-carbohydrate, low-fat (LF) diet in terms of their effect on endothelial function after 12 months in overweight and obese individuals	49 participants group 1 = reduced-energy diet, very low in carbohydrates and high in saturated fats (LC) group 2 = isocaloric diet, high in carbohydrates and low in fat (LF) (moderate energy restriction in both groups)	-significant reduction in blood pressure in each group after 12 months: -reduced blood pressure in the LC group = −14 ± 2 mmHg SBP and −6 ± 2 mmHg DBP -reduced blood pressure in the LF group = −15 ± 3 mmHg SBP and −8 ± 2 mmHg DBP -body weight loss in both groups (LC −14.9 ± 2.1 kg, LF −11.5 ± 1.5 kg) -improved pulse wave velocity (PWV) in both groups (LC −1.4 ± 0.6 m s(−1), LF −1.5 ± 0.6 m s(−1)), -improved flow-mediated dilatation (FMD) in LC (5.7 ± 0.7% to 3.7 ± 0.5%), no change in LF (5.9 ± 0.5% to 5.5 ± 0.7%)
2009 [193]	To compare a very-low-carbohydrate, high-saturated-fat diet (LC) against a high-carbohydrate, low-fat diet (LF) after 1 year in individuals with abdominal obesity and at least 1 additional metabolic syndrome risk factor	118 participants group 1 = very-low-carbohydrate, high-saturated-fat diet (LC) group 2 = high-carbohydrate, low-fat diet (LF)	-in the LC group, systolic blood pressure reduced from 132.7 ± 2.3 mmHg to 118.9 ± 2.0 mmHg and diastolic blood pressure reduced from 72.3 ± 1.8 mmHg to 66.0 ± 2.0 mmHg -in the LF group, SBP reduced from 135.2 ± 2.1 mmHg to 120.6 ± 2.9 mmHg and DBP reduced from 77.1 ± 1.8 mmHg to 69.2 ± 1.7 mmHg -in both groups, similar improvements in body weight, glucose, insulin, insulin resistance and CRP were observed after 12 months
2008 [194]	To compare the effects of a very-low-carbohydrate, high-fat (VLCHF) diet against a high-carbohydrate, low-fat (HCLF) diet in terms of effect on weight loss and cardiovascular disease (CVD) risk in adults with abdominal obesity	88 participants group 1 = very-low-carbohydrate, high-fat (VLCHF) diet group 2 = high-carbohydrate, low-fat (HCLF)	-in the VLCHF group, SBP reduced from 133.1 ± 14.4 mmHg to 120.8 ± 11.5 mmHg, and DBP reduced from 73.6 ± 11.6 mmHg to 69.0 ± 11.7 mmHg -in the HCLF group, SBP reduced from 136.1 ± 12.6 mmHg to 125.2 ± 15.8 mmHg and DBP reduced from 77.8 ± 10.1 mmHg to 72.3 ± 9.01 mmHg -similar body weight, CRP, fasting glucose and insulin level improvements accompanied by body weight loss were reported in both diets
2003 [195]	To assess the effect of a very low carbohydrate diet on body composition and cardiovascular risk factors	53 participants (females) group 1 = ad libitum, very low-carbohydrate diet group 2 = calorie-restricted diet with 30% of calories coming from fats	-normal blood pressure in both groups at the beginning of the study, after 3 months, and after 6 months -in the low-carbohydrate group, the initial BP values were 116/79 (3.23/2.69) mmHg, and changed to 112/72 (2.36/2.06) mmHg after 3 months, and to 114/74 (2.82/2.23) mmHg after 6 months -in the low-fat group, the initial BP values were 115/75 (2.47/1.99) mmHg, and changed to 116/75 (2.01/1.79) mmHg after 3 months, and to 113/74 (2.41/1.62) mmHg after 6 months -the low-carbohydrate group reported greater body weight loss (8.5 ± 1.0 kg vs. 3.9 ± 1.0 kg) and body fat loss (4.8 ± 0.67 kg vs. 2.0 ± 0.75 kg) -in both groups, lipid, fasting glucose and insulin levels improved

HKD—Healthy Ketogenic Diet; ERD—Energy-Restricted Diet; AKD—Asian Ketogenic Diet; VLCKD—Very Low-Calorie Ketogenic Diet; KD—Ketogenic Diet; BLC—Balanced Low-Caloric Diet; LFD—Low-Fat Diet; VLC—Very-Low Carbohydrate; LCKD—Low-Calorie Ketogenic Diet; DASH—Dietary Approaches to Stop Hypertension; VAT—Visceral Adipose Tissue; BMC—Bone Mineral Content; NKD—Non-Ketogenic Diet; WPG—Whey Protein Group; VPG—Vegetable Protein Group; APG—Animal Protein Group; HC—High Carbohydrate; O—Orlistat; VLF—Very-Low Fat; HUF—High Unsaturated Fat; PWV—Pulse Wave Velocity; FMD—Flow-Mediated Dilatation; VLCHF—Very-Low Carbohydrate, High-FAT Diet; HCLF—High-Carbohydrate, Low-Fat; CPAP—Continuous Positive Airway Pressure; OSAS—Obstructive Sleep Apnoea Syndrome; RAA—Renin–Angiotensin–Aldosterone system; KS—Ketone Salts; PL—Placebo; BMI—Body Mass Index; HbA1c—Haemoglobin A1c; HOMA—Homeostasis Model Assessment; SBP—Systolic Blood Pressure; DBP—Diastolic Blood Pressure; HDL—High-Density Lipoprotein; LDL—Low-Density Lipoprotein; AST—Aspartate Aminotransferase.

## Data Availability

No new data were created or analyzed in this study. Data sharing is not applicable to this article.
